# Standardized Patient Simulation Using SBIRT (Screening, Brief Intervention, and Referral for Treatment) as a Tool for Interprofessional Learning

**DOI:** 10.15766/mep_2374-8265.10955

**Published:** 2020-09-11

**Authors:** Janelle Clauser, Barbara B. Richardson, Tamara Odom-Maryon, Donna Mann, Megan N. Willson, Patricia L. Hahn, Janet Purath, Erica Tuell, Catrina R. Schwartz, Dawn DePriest

**Affiliations:** 1 Clinical Associate Professor, Department of Internal Medicine, University of Washington School of Medicine; 2 Clinical Associate Professor, Department of Medical Education and Clinical Sciences, Elson S. Floyd College of Medicine, Washington State University; 3 Research Professor, College of Nursing, Washington State University; 4 Associate Dean, College of Health Science and Public Health, and Associate Professor, Department of Occupational Therapy, Eastern Washington University; 5 Clinical Associate Professor, Department of Pharmacotherapy, College of Pharmacy and Pharmaceutical Sciences, Washington State University; 6 Senior Lecturer, Department of Family Medicine, University of Washington School of Medicine; 7 Associate Professor, College of Nursing, Washington State University; 8 Research Study Coordinator, College of Nursing, Washington State University; 9 Pharmacist, Pharmacy Management, Medication Review, Inc.; 10 Clinical Assistant Professor, College of Nursing, Washington State University

**Keywords:** SBIRT, Substance Use, Interprofessional, Multidisciplinary, Simulation, Standardized Patients, Case-Based Learning, Clinical Teaching/Bedside Teaching, Flipped Classroom, Problem-Based Learning, Editor's Choice

## Abstract

**Introduction:**

Substance misuse is a critical social and health care issue, and learning how to effectively screen for misuse and perform a brief intervention is useful for all health care professions. As an intercollegiate, interprofessional group, we developed a mechanism for delivering interprofessional education (IPE) using SBIRT (screening, brief intervention, and referral for treatment) as a tool to identify potential substance misuse.

**Methods:**

A total of 1,255 students from nursing, pharmacy, medicine, physician assistant, social work, dietetics, and occupational therapy programs participated in the training and evaluation of this IPE experience over 2 academic years. The training incorporated asynchronous SBIRT training, in-person student role-plays, and a standardized patient (SP) interaction.

**Results:**

A significant majority of participants indicated that this IPE experience enhanced their interprofessional skills (91%), was useful for interprofessional development (79%), was relevant to their career (92%), and would benefit their clients (93%). Faculty debrief sessions supported the efficacy of SBIRT as a platform for IPE.

**Discussion:**

Students believed that utilizing SBIRT as an interprofessional learning experience enhanced their overall educational experience and assisted with developing interprofessional relationships and that team-based care would lead to improved patient outcomes. Faculty found this learning activity to be effective in developing student insight regarding future professional peers and patient interview skill development through role-plays with peers and SPs.

## Educational Objectives

By the end of this activity, learners will be able to:

1. Describe screening, brief intervention, and referral for treatment (SBIRT) as a valuable tool for multiple health professions for identifying risk of substance misuse and providing a brief, point-of-care intervention.
2. Identify their role in the SBIRT process and compare roles with those of other health professional students (nursing, pharmacy, medicine, social work, occupational therapy, physician assistant, and dietetics).
3. Apply an evidence-based tool (SBIRT) to demonstrate a brief intervention in an interprofessional setting with a standardized patient.
4. Demonstrate giving and receiving timely, instructive feedback between team members regarding their interactions with a simulated patient.

## Introduction

Currently, substance misuse is a widespread social and health care issue with implications for all health care professions. The screening, brief intervention, and referral to treatment (SBIRT) technique is an evidence-based tool to identify potential misuse and prevent abuse of alcohol and/or illicit drugs. SBIRT was selected for this interprofessional activity because it incorporates common curricular content, including team-based patient-centered care, motivational interviewing skills, and population health and prevention.

Interprofessional education (IPE) programs require strong faculty commitment and a dedication to ongoing collaboration.^1^ The goal of IPE is to prepare workforce-ready health care team members who practice collaboratively in order to improve health outcomes.^2^ In response to the growing complexity of care, as well as accreditation requirements, health professions programs now integrate teamwork and communication content in their curricula. In order to create meaningful IPE learning opportunities, educators strive to design, implement, and evaluate learning opportunities that simulate patient care scenarios students will likely encounter in clinical settings.

The resource described here drew from lessons learned through a previous interprofessional Health Service and Research Administration–funded program that included faculty development, implementation of an interprofessional team-based program to teach collaborative practice skills using standardized patients (SPs), and multiple assessment and evaluation strategies to provide formative feedback to students.^3^ Using SBIRT as the vector for IPE, the goal of this program was to provide a meaningful interprofessional learning activity for students (*N* = 1,255) in medicine, nursing (BSN and DNP), pharmacy, physician assistant, social work, dietetics, and occupational therapy programs.

Other *MedEdPORTAL* publications have shared SBIRT cases for medical resident training using SPs^4^ or using SBIRT to facilitate interprofessional instruction of postgraduate health care professionals.^5,6^ In this module, we offer a guide for presession independent learning followed by SBIRT cases for a single in-person session of peer role-play practice and SP interactions, with students from seven different health professions as the target learners.

## Methods

### Design

This project was reviewed by the Washington State University Institutional Review Board (the host university), which determined the project satisfied the criteria for exempt research.

We designed this SBIRT educational module for teaching interprofessional student learners from concept to skills application. We assembled an interprofessional team comprising medicine, nursing (BSN and DNP), pharmacy, physician assistant, social work, dietetics, and occupational therapy faculty to design, implement, and evaluate the educational module and learning objectives (Appendix A). Student participants represented the same seven professions. These programs were included because they were professions likely to use motivational interviewing skills, specifically SBIRT, in collaborative care practice settings. We chose substance misuse as the focus of the program because of the ubiquitous impact of alcohol and illicit drugs in all health care settings and all communities. After evaluating numerous options, we selected SBIRT because it was evidence based and applied strategies of preventive care, motivational interviewing, and team-based patient-centered care. All faculty who developed and delivered content completed a 4-hour SBIRT training provided by a certified SBIRT educator.

For student participants, the educational module consisted of a required 2-hour online informational component (completed at any point prior to the live session) and a 2-hour in-person application session. Participation was required as a class assignment in each profession-specific program.

#### Preclass component

A subset of faculty reviewed available online programs for quality and applicability across multiple disciplines, as well as ease of registration and use. Trainings from Medscape (which later became unavailable) and SBIRT Oregon were selected.^7^ These two trainings were similar in defining SBIRT, introducing screening tools for identifying drug and alcohol misuse, demonstrating a brief intervention, and discussing impacts and benefits of using SBIRT. Prior to the in-person session, all students were required to complete the online preclass component and watch a video^8^ (Appendix D) that provided an overview of substance misuse, produced by MU-ADEPT (the University of Missouri's Alcohol and Drug Education for Prevention and Treatment program) and funded by the Substance Abuse and Mental Health Services Administration (SAMHSA). Instructions for the preclass component were included in program-specific course syllabi and emailed to students prior to the in-person session (Appendix C).

#### In-person application session

The 2-hour live session consisted of three main components. The first component was viewing a brief video demonstration from SBIRT Colorado^9^ (Appendix F) to review and consolidate knowledge gained from preclass preparation. Next was a peer role-play for students to practice SBIRT skills and share peer feedback (Appendices L–T). The third component was the SP interaction to apply skills and receive SP feedback (Appendices U–X). Faculty used the same logistics format (Appendices B and G), informational slides (Appendix H), and general script (Appendix I) at each in-person session.

### Case Development

Case development followed three principles for success: connecting relevance to clinical practice,^10^ faculty development for a shared IPE vision, and equality among all faculty participants.^11^ All cases encompassed patients of varying ages, medical settings, and reasons for seeking medical care. Alcohol use was the common theme for each scenario.

We adapted three cases for the peer role-play from existing SBIRT resources.^12^ Each case contained a brief introduction, a summary of the setting and provider's role, background information for the patient's role including a completed screening questionnaire, and a feedback rubric for the observer role. The students worked in interprofessional triads; every student took a turn enacting each role (patient, provider, observer) over the course of three cases. Case roles were color coded by rounds so students could more easily follow the format (Appendices L–T). Students also received an agenda (Appendix K), copies of a screening tool (Appendix E), and an SBIRT pocket card (Appendix J).

For the SP interaction, each student remained in the same triad and rotated turns implementing the SBIRT process with the SP and providing peer-to-peer feedback. We adapted three cases for SPs based on SAMHSA training materials (Appendices U–X include two of these); each triad received one of the two patient backgrounds, which remained consistent. We created scripts of possible behavioral responses, which the SP changed for each of the three students in the triad. These responses included amicable to change, minimizing the problem, or resistant to change. All project faculty reviewed and edited each case for applicability across disciplines.

### SP Training

For this project, an SP was defined as an individual trained to portray a real patient in order to simulate a set of symptoms or problems used for health care education.^13^ All SPs were recruited and trained by the same experienced SP educator. As per the Association of Standardized Patient Educators' Standards of Best Practice recommendations, a psychologically and physically safe working environment was provided.^14^ The SPs reviewed the scripts (Appendices U and V or W and X, depending on which case they were assigned) and attended an orientation and practice session at least 2 weeks prior to the activity. An additional training session occurred immediately prior to the activity and included final review of the script, as well as guidance on focused feedback methods. SPs were coached to start with an open-ended question to begin feedback (i.e., “How did you think it went?”) followed by review of targeted feedback items from the student observer checklist (Appendix L) and ending with reflection on how they could collaborate with other professions with similar patients. SP training also included instructions about providing time-outs if students grew flustered or had an emotional reaction to the content. During the SP/student interactions, faculty rotated to each triad to observe interactions and troubleshoot if needed. Any suggestions for improving SP interactions with students were given to the SP educator, who communicated directly with the SPs. SPs were also provided an opportunity to debrief with the SP educator following each session. Suggestions for improvements were addressed by the faculty team at regularly scheduled planning and debriefing sessions.

### Concluding the Session

After the SP interaction, students reconvened in the large group for debriefing. The debrief focused on the interprofessional collaborative experience (Appendix H, slide 7 notes). We asked students to complete an electronic survey at the end of the session.

### Assessment of Student Performance and Perceptions

#### Program evaluation

All students were asked to complete a survey at the conclusion of the training (Appendix Y). The survey included 17 questions from the Center for Substance Abuse Training (as required by the SAMHSA grant), 10 questions from a modified Student Perceptions of Physician-Pharmacist Interprofessional Clinical Education (SPICE) tool,^3,15^ and seven questions developed by our team. Two of the four Interprofessional Education Collaborative (IPEC) core competencies (Roles and Responsibilities, Teams and Teamwork) were addressed by the SPICE tool, so we added two questions regarding the remaining two IPEC core competencies (Communication and Collaboration, Values and Ethics).^16,17^ One question asked students to rate SBIRT as an effective interprofessional learning activity, and one question addressed how useful the activity was to their interprofessional development.

Finally, students completed three open-ended qualitative questions:

1. Based on your experience, what do you believe are the benefits of an interprofessional team approach to caring for patients with substance abuse behaviors?
2. What about the training was most useful in supporting your work responsibilities?
3. How can Center for Substance Abuse Training improve its training?

We examined these responses qualitatively using principles of inductive reasoning—a common approach in qualitative analysis that allows researchers to gain insights beyond those possible through quantitative methods alone.^18^ The lead qualitative researcher monitored the process for quality and rigor by employing reflexivity and triangulation. Reflexivity acknowledges the presence of bias and seeks to mitigate its influence during the data-analysis process.^19,20^ Triangulation is a methodological check on the validity of the data analysis and was accomplished by triangulating iterative coding phases among four qualitative researchers. Conceptual codes and subcodes^21^ were developed as an outcome of this process.

Qualtrics Research Suite software was used to deploy the anonymous, web-based electronic survey. Quantitative data were summarized as frequencies (percentages) for each item.

#### Formative feedback

In addition to the feedback from SPs (described in SP Training, above), students provided peer-to-peer verbal feedback immediately following each role-play and SP interaction using the observational rubric (Appendix L). Observer forms were not collected or analyzed. The importance of giving specific constructive suggestions for improvement was reviewed with participants prior to the role-play activity. Faculty circulated and provided additional guidance on feedback where needed.

## Results

Over 2 academic years from 2016 to 2018, 1,255 students participated in the interprofessional activity and completed any survey questions. Of these, 985 (79%) found the activity very useful/useful to their interprofessional development (learning objectives 1 and 4). Students were very satisfied/satisfied with the training regarding the following:

• The training enhanced my skills in this topic area (91%, learning objective 1).
• I expect to use the information gained from this training (91%, learning objectives 2 and 3).
• I expect this training to benefit my clients (93%, learning objective 3).

Students' perceived value of working with interprofessional teams was high following the training activity (Table 1).

**Table 1.**
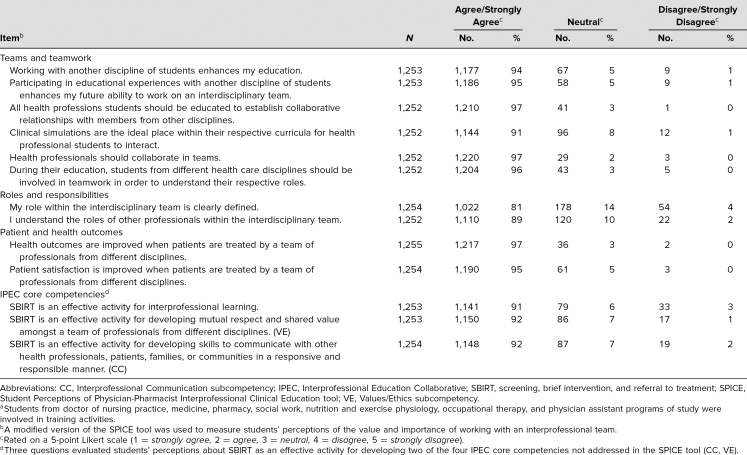
Students' Perceptions of the Value/Importance of Working With an Interprofessional Team Following an Interprofessional Training Activity Using the SBIRT Technique (*N* = 1,258)^a^

Qualitative data analysis resulted in seven themes across the three categories of usefulness (learning objectives 1 and 3), evaluation (learning objective 1), and benefit (learning objectives 2 and 4). Table 2 sets out the categories and their associated themes. Overall, students were highly satisfied with the interprofessional learning activity and interactions with an SP. Students believed this type of training prepared them to work collaboratively in teams and supported their comfort level in approaching difficult conversations with patients. For representative student comments arranged by category and theme, see Table 3.

**Table 2.**
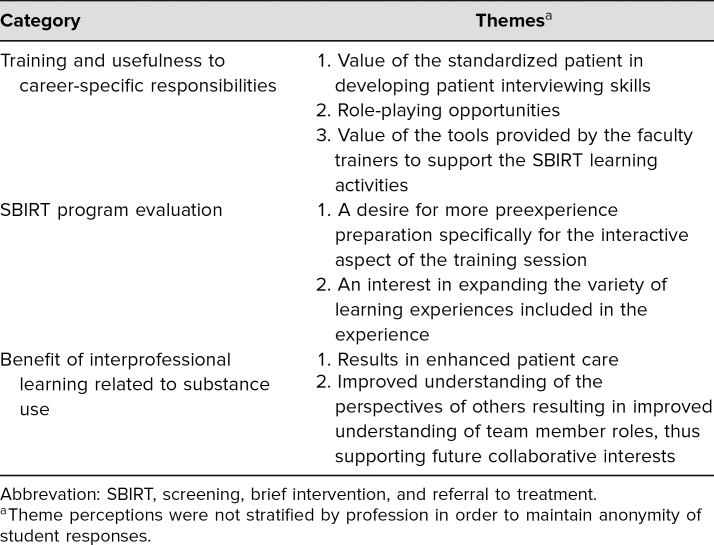
Students' Perceptions of the Value/Importance of an Interprofessional Team Learning Experience Using the SBIRT Technique

**Table 3.**
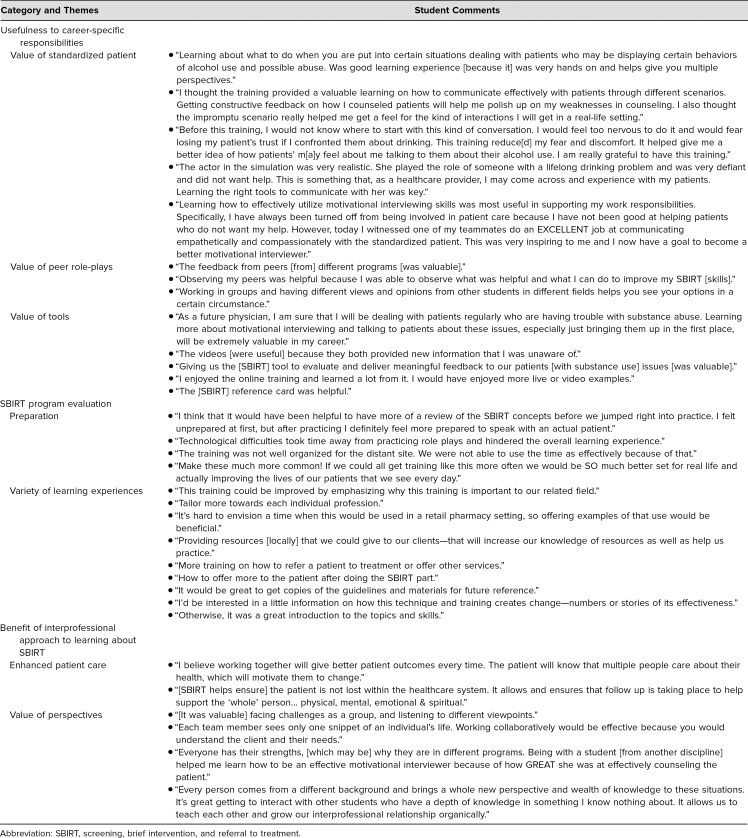
Students' Comments Regarding of the Value/Importance/Effectiveness of an Interprofessional Team Learning Experience Using the SBIRT Technique

A discipline-specific evaluation of student performance and perceptions of the potential impact of the training using a smaller cohort of students has been described elsewhere.^22^

## Discussion

Growing interest in IPE activities stems from demonstrated improvements in patient safety^23^ as well as accreditation requirements and the increasing recognition that health professional students benefit from engaging in cooperative work early in their training. We successfully used SBIRT as a model for SP simulation and realistic interprofessional collaboration.

We developed our cases with input and review from professionals in all seven participating disciplines; thus, all student participants were able to fully engage as equal members of the team in the SP interaction. The students learned about substance use and SBIRT from preclass preparation materials (Appendix D and SBIRT Oregon^7^ materials) and had varying levels of prior experience with SPs. Students highly valued this SBIRT IPE learning experience as indicated by postexperience results.

The activity was very successful in students practicing interprofessional collaboration as well as the value of using a standardized process such as SBIRT. The vast majority of participants said that the activity enhanced their skills (91%), that they expected to use the information (93%), and that it would benefit their clients (93%). This activity gave students an opportunity to gain skills they found useful to their practice with current and future patients.

With three universities and seven disciplines involved, scheduling the interactive sessions was complex, but all disciplines successfully embedded the activity into an existing course. Interprofessional faculty collaboration was critical; this included early communication regarding logistics so each faculty could plan their courses accordingly, as well as regular meetings to coordinate the experience and then engage in ongoing assessment for program improvements. Numerous small-group sessions at varying times of day were needed to accommodate all disciplines because of highly diverse student schedules.

Strengths of this module include the focus on previsit interprofessional collaborative dialogue and planning, which allowed students to discuss and decide what each member's roles and responsibilities would be. Taking turns interviewing and counseling the SP helped develop each student's communication skills as well as the ability to observe other team members and provide real-time feedback using a rubric.

Potential limitations to this activity include the need for multiple health professions to be present, which may be a challenge, especially in more rural educational settings. SP simulations were done in triads, so this activity could be done with as few as two to three different health professions. In low-resource areas where programs may not have availability or funding for SP actors, faculty or students can use the scripts to play these roles. We included a videoconferencing option for students in rural educational sites; however, both students and faculty agreed that the technological frustrations outweighed the benefits of this component. A further limitation was that due to the small number of students in some professions, we did not identify themed perceptions by profession in order to protect participants' anonymity.

In future iterations of this activity, we would incorporate students describing their roles and those of other professions in screening for substance misuse during the icebreaker activity (Appendix H, slide 2). This activity represents an SP simulation that can be used by any program that desires to have a high-quality, single-session, collaborative interprofessional experience, with special focus on substance use screening and motivational interviewing.

## Appendices

Educational Objectives.docxAdministrative Instructions Prior to Session.docxStudent Overview of SBIRT Components - Email Prior.docxStudent Prep - ADEPT Video.mp4AUDIT Screening Tool - Email and Print.docxDemonstration - SBIRT Colorado.mp4Faculty Overview and Agenda.docxSBIRT Slides for Live Session.pptxFaculty Script for Slide Presentation.docxSBIRT Pocket Card - Print.pdfStudent Agenda - Print.docxPeer Role-Play Case 1-Print ORANGE-Observer.docxPeer Role-Play Case 1-Print ORANGE-Patient.docxPeer Role-Play Case 1-Print ORANGE-Provider.docxPeer Role-Play Case 2-Print BLUE-Observer.docxPeer Role-Play Case 2-Print BLUE-Patient.docxPeer Role-Play Case 2-Print BLUE-Provider.docxPeer Role-Play Case 3-Print GREEN-Observer.docxPeer Role-Play Case 3-Print GREEN-Patient.docxPeer Role-Play Case 3-Print GREEN-Provider.docxSP Case Jamie Quimby.docxSP AUDIT Screen Jamie Quimby.pdfSP Case Pat Stewart.docxSP AUDIT Screen Pat Stewart.pdfEvaluation Tool.docx
All appendices are peer reviewed as integral parts of the Original Publication.
