# Supplementary material for: Standardized Patient Simulation Using SBIRT (Screening, Brief Intervention, and Referral for Treatment) as a Tool for Interprofessional Learning
Source: MedEdPORTAL. 2020 Sep 11;16:10955. doi: 10.15766/mep_2374-8265.10955 (PMC7485913; doi:10.15766/mep_2374-8265.10955)
Supplement: Supplementary file 1 — Educational Objectives.docxAdministrative Instructions Prior to Session.docxStudent Overview of SBIRT Components - Email Prior.docxStudent Prep - ADEPT Video.mp4AUDIT Screening Tool - Email and Print.docxDemonstration - SBIRT Colorado.mp4Faculty Overview and Agenda.docxSBIRT Slides for Live Session.pptxFaculty Script for Slide Presentation.docxSBIRT Pocket Card - Print.pdfStudent Agenda - Print.docxPeer Role-Play Case 1-Print ORANGE-Observer.docxPeer Role-Play Case 1-Print ORANGE-Patient.docxPeer Role-Play Case 1-Print ORANGE-Provider.docxPeer Role-Play Case 2-Print BLUE-Observer.docxPeer Role-Play Case 2-Print BLUE-Patient.docxPeer Role-Play Case 2-Print BLUE-Provider.docxPeer Role-Play Case 3-Print GREEN-Observer.docxPeer Role-Play Case 3-Print GREEN-Patient.docxPeer Role-Play Case 3-Print GREEN-Provider.docxSP Case Jamie Quimby.docxSP AUDIT Screen Jamie Quimby.pdfSP Case Pat Stewart.docxSP AUDIT Screen Pat Stewart.pdfEvaluation Tool.docx [file mep_2374-8265.10955-s001.zip › A. Educational Objectives.docx]

Educational Objectives

By the end of this session, learners will be able to:

1. Describe SBIRT as a valuable tool for multiple health professions for identifying risk of substance misuse and providing a brief, point of care intervention.

*Maps to IPEC Core Competency CC1: Choose effective communication tools and techniques, including information systems and communication technologies, to facilitate discussions and interactions that enhance team function.*

2. Identify their role in the SBIRT process and compare roles with those of other health professional students (nursing, pharmacy, medicine, social work, occupational therapy, physician assistant, and dietetics).

*Maps to IPEC Core Competencies RR1: Communicate one’s roles and responsibilities clearly to patients, families, community members, and other professionals; and RR2: Recognize one’s limitations in skills, knowledge, and abilities.*

3. Apply an evidence-based tool (SBIRT) to demonstrate a brief intervention in an interprofessional setting with a standardized patient.

*Maps to IPEC Core Competencies RR9: Use unique and complementary abilities of all members of the team to optimize health and patient care; and TT3: Engage health and other professionals in shared patient-centered and population focused problem-solving.*

4. Demonstrate giving and receiving timely, instructive feedback between team members regarding their interactions with a simulated patient.

*Maps to IPEC Core Competency CC5: Give timely, sensitive, instructive feedback to others about their performance on the team, responding respectfully as a team member to feedback from others.*

*Objectives are mapped to Interprofessional Education Collaborative (IPEC) 2016 core competencies^17^ as above. CC=Interprofessional Communication Sub-competency*

*RR=Roles/Responsibilities Sub-competency*

*TT=Team/Teamwork Sub-competency*
