# Supplementary material for: Standardized Patient Simulation Using SBIRT (Screening, Brief Intervention, and Referral for Treatment) as a Tool for Interprofessional Learning
Source: MedEdPORTAL. 2020 Sep 11;16:10955. doi: 10.15766/mep_2374-8265.10955 (PMC7485913; doi:10.15766/mep_2374-8265.10955)
Supplement: Supplementary file 1 — Educational Objectives.docxAdministrative Instructions Prior to Session.docxStudent Overview of SBIRT Components - Email Prior.docxStudent Prep - ADEPT Video.mp4AUDIT Screening Tool - Email and Print.docxDemonstration - SBIRT Colorado.mp4Faculty Overview and Agenda.docxSBIRT Slides for Live Session.pptxFaculty Script for Slide Presentation.docxSBIRT Pocket Card - Print.pdfStudent Agenda - Print.docxPeer Role-Play Case 1-Print ORANGE-Observer.docxPeer Role-Play Case 1-Print ORANGE-Patient.docxPeer Role-Play Case 1-Print ORANGE-Provider.docxPeer Role-Play Case 2-Print BLUE-Observer.docxPeer Role-Play Case 2-Print BLUE-Patient.docxPeer Role-Play Case 2-Print BLUE-Provider.docxPeer Role-Play Case 3-Print GREEN-Observer.docxPeer Role-Play Case 3-Print GREEN-Patient.docxPeer Role-Play Case 3-Print GREEN-Provider.docxSP Case Jamie Quimby.docxSP AUDIT Screen Jamie Quimby.pdfSP Case Pat Stewart.docxSP AUDIT Screen Pat Stewart.pdfEvaluation Tool.docx [file mep_2374-8265.10955-s001.zip › B. Administrative Instructions Prior to Session.docx]

Administrative instructions prior to session:

1. From your pool of participating student names, arrange them in groups of three (triads) preferably each from a different discipline within the triad.

2. Assign each triad a number. Then assign each student within the triad either A, B, or C.

Example: Triad 1 has 3 students, 1A, 1B, 1C; Triad 2 has 3 students, 2A, 2B, 2C

If you have a number not divisible by 3, you can use a group of 2 and have them alternate roles

(or have a group of 4 but then students will not get to play all 3 roles)

3. Reserve rooms:

- For first half of session and ending debrief: 1 large room

- For standardized patient interactions: 1 small room per triad

4. Recruit standardized patients:

- Recruit one standardized patient per student triad

- Share scripts with standardized patients ahead of time – (appendices U and V for Jamie case, or appendices W and X for Pat case)

- Print “completed” SP AUDIT screens (appendix V or X) – provide SP copy on day of live session

5. Assemble student packets (one folder per student): label each folder with student letter + number

For each **TRIAD** of students, print:

| # of copies to print **per triad** | Appendix | File name | Paper Color to use | Place in which folder(s) |
| --- | --- | --- | --- | --- |
| 3 | E | Audit Screening Tool | White | 1 each in all folders |
| 3 | J | SBIRT pocket card | White | 1 each in all folders |
| 3 | K | Student Agenda | White* | 1 each in all folders |
| 1 | L | Peer role play Case 1-ORANGE-Observer | Orange | A’s folders |
| 1 | M | Peer role play Case 1-ORANGE-Patient | Orange | B’s folders |
| 1 | N | Peer role play Case 1-ORANGE-Provider | Orange | C’s folders |
| 1 | O | Peer role play Case 2-BLUE-Observer | Blue | B’s folders |
| 1 | P | Peer role play Case 2-BLUE-Patient | Blue | C’s folders |
| 1 | Q | Peer role play Case 2-BLUE-Provider | Blue | A’s folders |
| 1 | R | Peer role play Case 3-GREEN-Observer | Green | C’s folders |
| 1 | S | Peer role play Case 3-GREEN-Patient | Green | A’s folders |
| 1 | T | Peer role play Case 3-GREEN-Provider | Green | B’s folders |

*consider using card stock for students to keep long-term

6. Contact participants ahead of time with the following attachments included:

| Appendix | File name | Description |
| --- | --- | --- |
| C | Student Overview of SBIRT Components | Description of expectations of pre-session knowledge prep and live session schedule |
| D | Student Prep – ADEPT video | Required student pre-session prep: video overview of SBIRT |
| E | AUDIT screening tool |  |
| F | Demonstration - SBIRT Colorado | Demonstration of Screening and Brief Intervention |

7. Create nametags that include: 1) participant name, 2) triad letter+number

8. Create sign-in sheet, if recording attendance is desired
