# Supplementary material for: Standardized Patient Simulation Using SBIRT (Screening, Brief Intervention, and Referral for Treatment) as a Tool for Interprofessional Learning
Source: MedEdPORTAL. 2020 Sep 11;16:10955. doi: 10.15766/mep_2374-8265.10955 (PMC7485913; doi:10.15766/mep_2374-8265.10955)
Supplement: Supplementary file 1 — Educational Objectives.docxAdministrative Instructions Prior to Session.docxStudent Overview of SBIRT Components - Email Prior.docxStudent Prep - ADEPT Video.mp4AUDIT Screening Tool - Email and Print.docxDemonstration - SBIRT Colorado.mp4Faculty Overview and Agenda.docxSBIRT Slides for Live Session.pptxFaculty Script for Slide Presentation.docxSBIRT Pocket Card - Print.pdfStudent Agenda - Print.docxPeer Role-Play Case 1-Print ORANGE-Observer.docxPeer Role-Play Case 1-Print ORANGE-Patient.docxPeer Role-Play Case 1-Print ORANGE-Provider.docxPeer Role-Play Case 2-Print BLUE-Observer.docxPeer Role-Play Case 2-Print BLUE-Patient.docxPeer Role-Play Case 2-Print BLUE-Provider.docxPeer Role-Play Case 3-Print GREEN-Observer.docxPeer Role-Play Case 3-Print GREEN-Patient.docxPeer Role-Play Case 3-Print GREEN-Provider.docxSP Case Jamie Quimby.docxSP AUDIT Screen Jamie Quimby.pdfSP Case Pat Stewart.docxSP AUDIT Screen Pat Stewart.pdfEvaluation Tool.docx [file mep_2374-8265.10955-s001.zip › C. Student Overview of SBIRT Components - Email Prior.docx]

**Student Overview**

**Screening, Brief Intervention and Referral to Treatment (SBIRT)**

**Interprofessional Learning Activity**

**Activity Components:**

1. **SBIRT Knowledge Preparation:** Pre-session preparation should take about 1 hour. Check with your course instructor for any additional requirements.

**Prior to the live session, students are required to:**

1. Read through this overview document in its entirety
2. Watch “Student Prep - ADEPT Video”
3. Watch “Demonstration – SBIRT Colorado” video
4. Review “AUDIT Screening Tool” and be prepared to use it in a clinical scenario
5. **SBIRT Live Session:** Two-hour interactive session, in student triads (see schedule below)

**Learning objectives:**

**At the conclusion of the interprofessional SBIRT training, students will be able to do the following.**

1. Describe SBIRT as a valuable tool for multiple health professions for identifying risk of substance misuse and providing a brief, point of care intervention.
2. Identify their role in the SBIRT process and compare roles with those of other health professional students (nursing, pharmacy, medicine, social work, occupational therapy, physician assistant, and dietetics).
3. Apply an evidence-based tool (SBIRT) to demonstrate a brief intervention in an interprofessional setting with a standardized patient.
4. Demonstrate giving and receiving timely, instructive feedback between team members regarding their interactions with a simulated patient.

**Screening, Brief Intervention and Referral to Treatment (SBIRT) Training Overview:**

***What is (SBIRT) and why is it important?*** SBIRT is a comprehensive public health core preventative strategy that addresses substance misuse and abuse which are prevalent nationwide. The screening allows quick assessment of the substance abuse severity and then ascertains the appropriate treatment level. The focus of SBIRT is to help patients focus on awareness and personal insight about their substance use and help then gauge their motivation to change behaviors. According to the 2013 National Drug Control Strategy data, evidence is indicating that SBIRT is impacting reductions in alcohol and drug use 6 months after receiving the SBIRT intervention, improving quality of life measures, such as employment and housing stability and is reducing risky behaviors, including unprotected sexual encounters.^+^ SBIRT is promoted by Substance Abuse and Mental Health Services Administration (SAMHSA) as an essential approach that should be integrated into primary care practices and that healthcare students and providers from all disciplines should be trained to use SBIRT with their patients.

***Why is this an interprofessional activity?*** Learning SBIRT alongside students from other healthcare programs provides a relevant platform for students to engage with each other as they develop important patient care skills for practice.

**SBIRT Training Goal:** To train health professions students in an interprofessional environment on SBIRT to ensure they are prepared to serve current and future patients regarding substance misuse and abuse.

**SBIRT Training Format:** The training includes two components: 1) an online educational module and 2) an interactive live session. On the day of your live SBIRT session, you will participate with a group of two other students from at least one other profession to practice SBIRT with guidance from faculty facilitators. Following the practice session, each small group will conduct a “real” SBIRT interaction with a standardized patient (paid actor). Students will complete a program evaluation. There may be other course requirements as defined by your instructor.

**SBIRT Live Session Schedule: Two-hour interactive session, in student triads**

**1^st^ hour:**

25 minutes: Welcome, session overview, SBIRT Review with video demonstration, opportunity for questions

40 minutes: Students break into pre-assigned triads and practice each of 3 SBIRT cases. Students will

rotate roles across the cases so that by the end of 3 cases each student has been the

patient, the provider or the observer. Materials will be provided during the session.

5-10 minutes: Short break, participants move to assigned standardized patient rooms

**2^nd^ hour:**

40 minutes: SBIRT interaction with standardized patient; students will give feedback to one another;

SP will provide feedback to all 3 at the end

5 minutes: Small groups move back to large room

15 minutes: Program evaluation survey, Large group debrief

**Questions about the SBIRT Training, course requirements or grading?** Please contact your course faculty.
