# Supplementary material for: Standardized Patient Simulation Using SBIRT (Screening, Brief Intervention, and Referral for Treatment) as a Tool for Interprofessional Learning
Source: MedEdPORTAL. 2020 Sep 11;16:10955. doi: 10.15766/mep_2374-8265.10955 (PMC7485913; doi:10.15766/mep_2374-8265.10955)
Supplement: Supplementary file 1 — Educational Objectives.docxAdministrative Instructions Prior to Session.docxStudent Overview of SBIRT Components - Email Prior.docxStudent Prep - ADEPT Video.mp4AUDIT Screening Tool - Email and Print.docxDemonstration - SBIRT Colorado.mp4Faculty Overview and Agenda.docxSBIRT Slides for Live Session.pptxFaculty Script for Slide Presentation.docxSBIRT Pocket Card - Print.pdfStudent Agenda - Print.docxPeer Role-Play Case 1-Print ORANGE-Observer.docxPeer Role-Play Case 1-Print ORANGE-Patient.docxPeer Role-Play Case 1-Print ORANGE-Provider.docxPeer Role-Play Case 2-Print BLUE-Observer.docxPeer Role-Play Case 2-Print BLUE-Patient.docxPeer Role-Play Case 2-Print BLUE-Provider.docxPeer Role-Play Case 3-Print GREEN-Observer.docxPeer Role-Play Case 3-Print GREEN-Patient.docxPeer Role-Play Case 3-Print GREEN-Provider.docxSP Case Jamie Quimby.docxSP AUDIT Screen Jamie Quimby.pdfSP Case Pat Stewart.docxSP AUDIT Screen Pat Stewart.pdfEvaluation Tool.docx [file mep_2374-8265.10955-s001.zip › E. AUDIT Screening Tool - Email and Print.docx]

**AUDIT Screening Tool**

| One drink equals: | 12 oz. beer | | 5 oz. wine | | | 1.5 oz. liquor (one shot) | |
| --- | --- | --- | --- | --- | --- | --- | --- |
| Place an X in one box that best describes your answer to each question. | | | | | | | |
| **In the past 12 months…** | **0** | **1** | | **2** | **3** | | **4** |
| 1. How often do you have a drink containing alcohol? | ○ Never | ○ Monthly or less | | ○ 2 to 4 times a month | ○ 2 to 3 times a week | | ○ 4 or more times a week |
| 2. How many drinks containing alcohol do you have on a typical day when you are drinking? | ○ 1 or 2 | ○ 3 or 4 | | ○ 5 or 6 | ○ 7 to 9 | | ○ 10 or more |
| 3. How often do you have 5 or more drinks on one occasion? | ○ Never | ○ Less than monthly | | ○ Monthly | ○ Weekly | | ○ Daily or almost daily |
| 4. How often during the last year have you found that you were not able to stop drinking once you had started? | ○ Never | ○ Less than monthly | | ○ Monthly | ○ Weekly | | ○ Daily or almost daily |
| 5. How often during the last year have you failed to do what was normally expected from you because of drinking? | ○ Never | ○ Less than monthly | | ○ Monthly | ○ Weekly | | ○ Daily or almost daily |
| 6. How often during the last year have you needed a first drink in the morning to get yourself going after a heavy drinking session? | ○ Never | ○ Less than monthly | | ○ Monthly | ○ Weekly | | ○ Daily or almost daily |
| 7. How often during the last year have you had a feeling of guilt or remorse after drinking? | ○ Never | ○ Less than monthly | | ○ Monthly | ○ Weekly | | ○ Daily or almost daily |
| 8. How often during the last year have you been unable to remember what happened the night before because of your drinking? | ○ Never | ○ Less than monthly | | ○ Monthly | ○ Weekly | | ○ Daily or almost daily |
| 9. Have you or someone else been injured because of your drinking? | ○ No |  | | ○ Yes, but not in the last year |  | | ○ Yes, during the last year |
| 10. Has a relative, friend, doctor, or other health care worker been concerned about your drinking or suggested you cut down? | ○ No |  | | ○ Yes, but not in the last year |  | | ○ Yes, during the last year |
| *Add scores for each column, then add across this row.* |  |  | |  |  | |  |
| **TOTAL** | | | | | | |  |

The **USAUDIT** is an adaptation of the WHO AUDIT to better identify individuals with risky patterns of alcohol consumption, as defined by the U.S. standard drink (14 grams) and recommended drinking limits (National Institute on Alcohol Abuse and Alcoholism [NIAAA], 2007), and those who may have an alcohol use disorder (AUD), according to the International Classification of Mental and Behavioral Diseases – Tenth Revision (ICD-10; WHO, 1993) and the Diagnostic and Statistical Manual of Mental Disorders – Fifth Edition (DSM-5; American Psychiatric Association [APA], 2013).

| **Guidelines for Interpretation for AUDIT** | | |
| --- | --- | --- |
| **Score** | **Risk Level** | **Intervention** |
| 0-6 (Female)  0-7 (Male) | Zone I | Feedback and alcohol education |
| 7-15 (Female)  8-15 (Male) | Zone II | Brief intervention |
| 16-19 | Zone III | Brief intervention plus brief therapy |
| 20-40 | Zone IV | Brief intervention plus referral to chemical dependency treatment |

Adapted from: Babor T, ‎et al. World Health Organization. (‎2001)‎. AUDIT: the Alcohol Use Disorders Identification Test: guidelines for use in primary health care. ‎2nd ed. World Health Organization.
