# Supplementary material for: Standardized Patient Simulation Using SBIRT (Screening, Brief Intervention, and Referral for Treatment) as a Tool for Interprofessional Learning
Source: MedEdPORTAL. 2020 Sep 11;16:10955. doi: 10.15766/mep_2374-8265.10955 (PMC7485913; doi:10.15766/mep_2374-8265.10955)
Supplement: Supplementary file 1 — Educational Objectives.docxAdministrative Instructions Prior to Session.docxStudent Overview of SBIRT Components - Email Prior.docxStudent Prep - ADEPT Video.mp4AUDIT Screening Tool - Email and Print.docxDemonstration - SBIRT Colorado.mp4Faculty Overview and Agenda.docxSBIRT Slides for Live Session.pptxFaculty Script for Slide Presentation.docxSBIRT Pocket Card - Print.pdfStudent Agenda - Print.docxPeer Role-Play Case 1-Print ORANGE-Observer.docxPeer Role-Play Case 1-Print ORANGE-Patient.docxPeer Role-Play Case 1-Print ORANGE-Provider.docxPeer Role-Play Case 2-Print BLUE-Observer.docxPeer Role-Play Case 2-Print BLUE-Patient.docxPeer Role-Play Case 2-Print BLUE-Provider.docxPeer Role-Play Case 3-Print GREEN-Observer.docxPeer Role-Play Case 3-Print GREEN-Patient.docxPeer Role-Play Case 3-Print GREEN-Provider.docxSP Case Jamie Quimby.docxSP AUDIT Screen Jamie Quimby.pdfSP Case Pat Stewart.docxSP AUDIT Screen Pat Stewart.pdfEvaluation Tool.docx [file mep_2374-8265.10955-s001.zip › G. Faculty Overview and Agenda.docx]

**Overview of Set-up for live session**

**Live Session Structure:**

- Two-hour session
- Best if total number of participants is divisible by 3
- Assign triads of students interprofessionally (with variety of disciplines in each triad)

**Faculty Facilitators:**

- Two or three per session
- Responsibilities:
  - Introduction:
    - Appendix H: “SBIRT Slides for Live Session”
    - Appendix I: “Faculty Script for Slide Presentation”
  - Facilitate the 1^st^ hour small group practice sessions
  - Facilitate large group debrief session at the end
  - One faculty should be available solely to handle logistics of the SP encounters (meeting SPs as they arrive and getting them to assigned rooms, etc)

**Standardized Patients:**

- One standardized patient (SP) per student triad in live session

**Audiovisual Equipment:**

- Projector setup or smartboard for introduction slides

**Room Requirements:**

- One large classroom for the introduction, small group practice, and debrief
- Smaller rooms for each standardized patient (SP) SBIRT encounter – one room per each triad

**Printed Materials:**

- Attendance roster
- Nametags
- Folders identifying group number (# of triads) and participant letter (A, B or C)
- Folder Contents: see appendix B for folder contents and instructions

**Optional Materials for Further Faculty Training (or Student Prep):**

- SBIRT Oregon: [www.sbirtoregon.org](http://www.sbirtoregon.org)
- Substance Abuse and Mental Health Services Administration (SAMHSA) SBIRT resources: <https://www.samhsa.gov/sbirt/resources>
- SAMHSA’s SBIRT case-based educational activity (via Medscape): <https://www.medscape.org/viewarticle/830331>

**Screening, Brief Intervention & Referral for Treatment (SBIRT)**

**Interprofessional Training Agenda**

**Detailed Faculty Version**

*See Appendix H (SBIRT Slides for Live Session) for introduction slides and Appendix I (Faculty Script for Slide Presentation) for talking points.*

3 min Welcome, Introduce faculty mentors (slide 1 of appendix H)

6 min Icebreaker – students talk in assigned triads (slide 2 of appendix H)

16 min Review of SBIRT (slides 3-5 of appendix H)

30 min Practice SBIRT (3 cases x 10 minutes = 30 minutes)

- In assigned interprofessional triads
- **Three rounds** to practice
  - For each round:
    - One student is the provider, one the patient, one the observer
    - 8 minutes for SBIRT interaction role play, then 2 min to provide constructive feedback to the student playing the provider
    - Roles rotate each round until each student has done all 3 roles

5 min Review what to expect for second hour (slide 6 of appendix H)

Move to small rooms for interaction with standardized patients

30 min SBIRT interaction with standardized patients (3 x 10 minutes = 30 minutes)

- Remain in same assigned triads
- **Three rounds** to practice
  - For each round:
    - 1 student will be the provider
    - 2 students will be the observers / timekeepers
  - Each student has 5-8 minutes for an SBIRT interview (total 15-20 min)
  - Three interviews are done consecutively by each student in triad (SP changes response per script)
  - Constructive feedback is shared at the end, between peers and from SP (total 6-15 min)

5 min Break and return to larger classroom

10 min Complete course evaluation (if desired) prior to large group debrief

10 min Large Group Debrief

**Sample debrief questions:**

1. Why do you think *interprofessional* SBIRT training is important?
2. What parts of the SBIRT training session helped you have a better understanding of the roles or responsibilities of another health profession?
3. How will you use the knowledge and skills you gained through the SBIRT training in your practice?
