# Supplementary material for: Standardized Patient Simulation Using SBIRT (Screening, Brief Intervention, and Referral for Treatment) as a Tool for Interprofessional Learning
Source: MedEdPORTAL. 2020 Sep 11;16:10955. doi: 10.15766/mep_2374-8265.10955 (PMC7485913; doi:10.15766/mep_2374-8265.10955)
Supplement: Supplementary file 1 — Educational Objectives.docxAdministrative Instructions Prior to Session.docxStudent Overview of SBIRT Components - Email Prior.docxStudent Prep - ADEPT Video.mp4AUDIT Screening Tool - Email and Print.docxDemonstration - SBIRT Colorado.mp4Faculty Overview and Agenda.docxSBIRT Slides for Live Session.pptxFaculty Script for Slide Presentation.docxSBIRT Pocket Card - Print.pdfStudent Agenda - Print.docxPeer Role-Play Case 1-Print ORANGE-Observer.docxPeer Role-Play Case 1-Print ORANGE-Patient.docxPeer Role-Play Case 1-Print ORANGE-Provider.docxPeer Role-Play Case 2-Print BLUE-Observer.docxPeer Role-Play Case 2-Print BLUE-Patient.docxPeer Role-Play Case 2-Print BLUE-Provider.docxPeer Role-Play Case 3-Print GREEN-Observer.docxPeer Role-Play Case 3-Print GREEN-Patient.docxPeer Role-Play Case 3-Print GREEN-Provider.docxSP Case Jamie Quimby.docxSP AUDIT Screen Jamie Quimby.pdfSP Case Pat Stewart.docxSP AUDIT Screen Pat Stewart.pdfEvaluation Tool.docx [file mep_2374-8265.10955-s001.zip › H. SBIRT Slides for Live Session.pptx]

## Slide 1
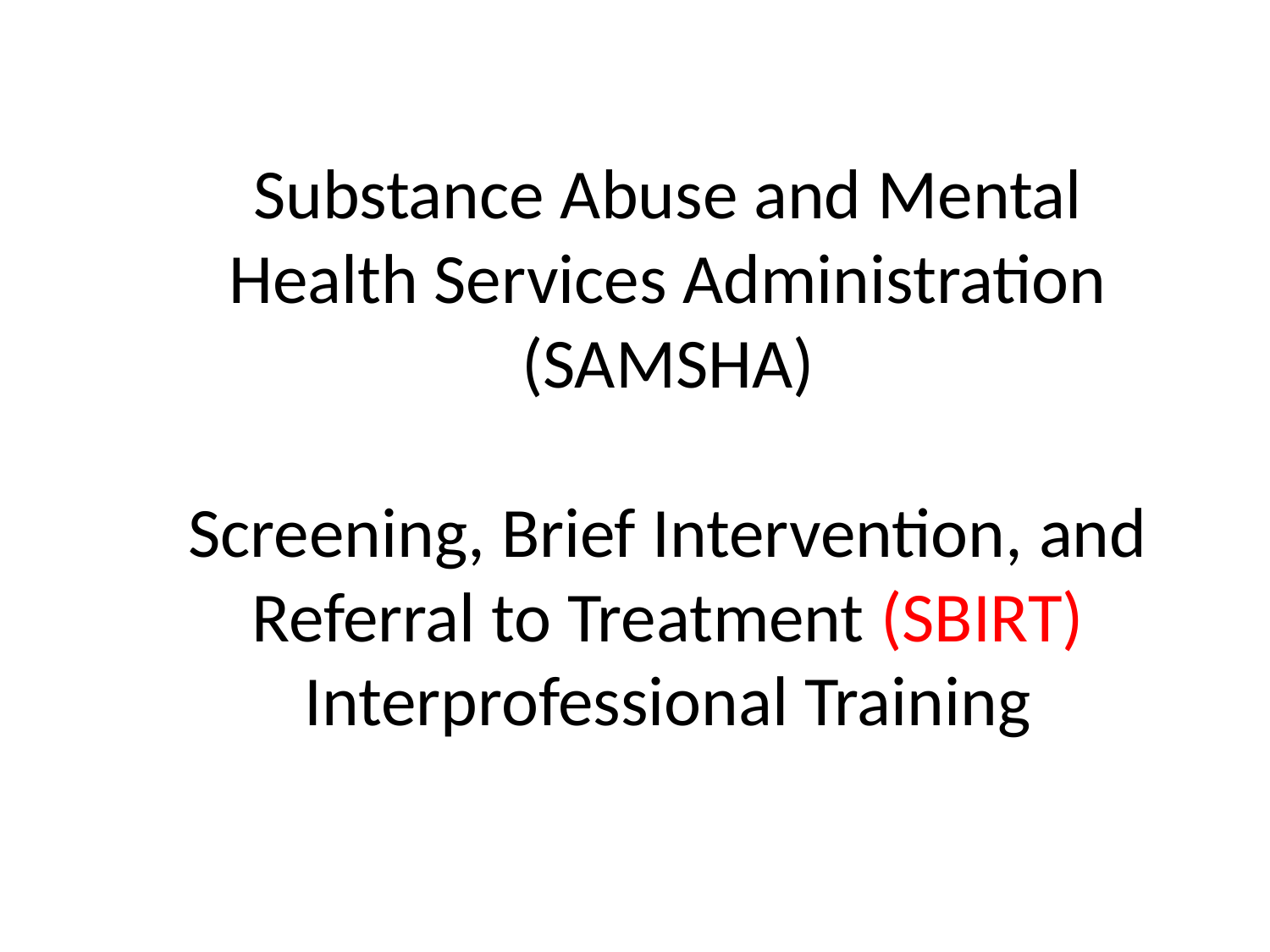

Substance Abuse and Mental Health Services Administration (SAMSHA)
Screening, Brief Intervention, and Referral to Treatment (SBIRT) Interprofessional Training

## Slide 2
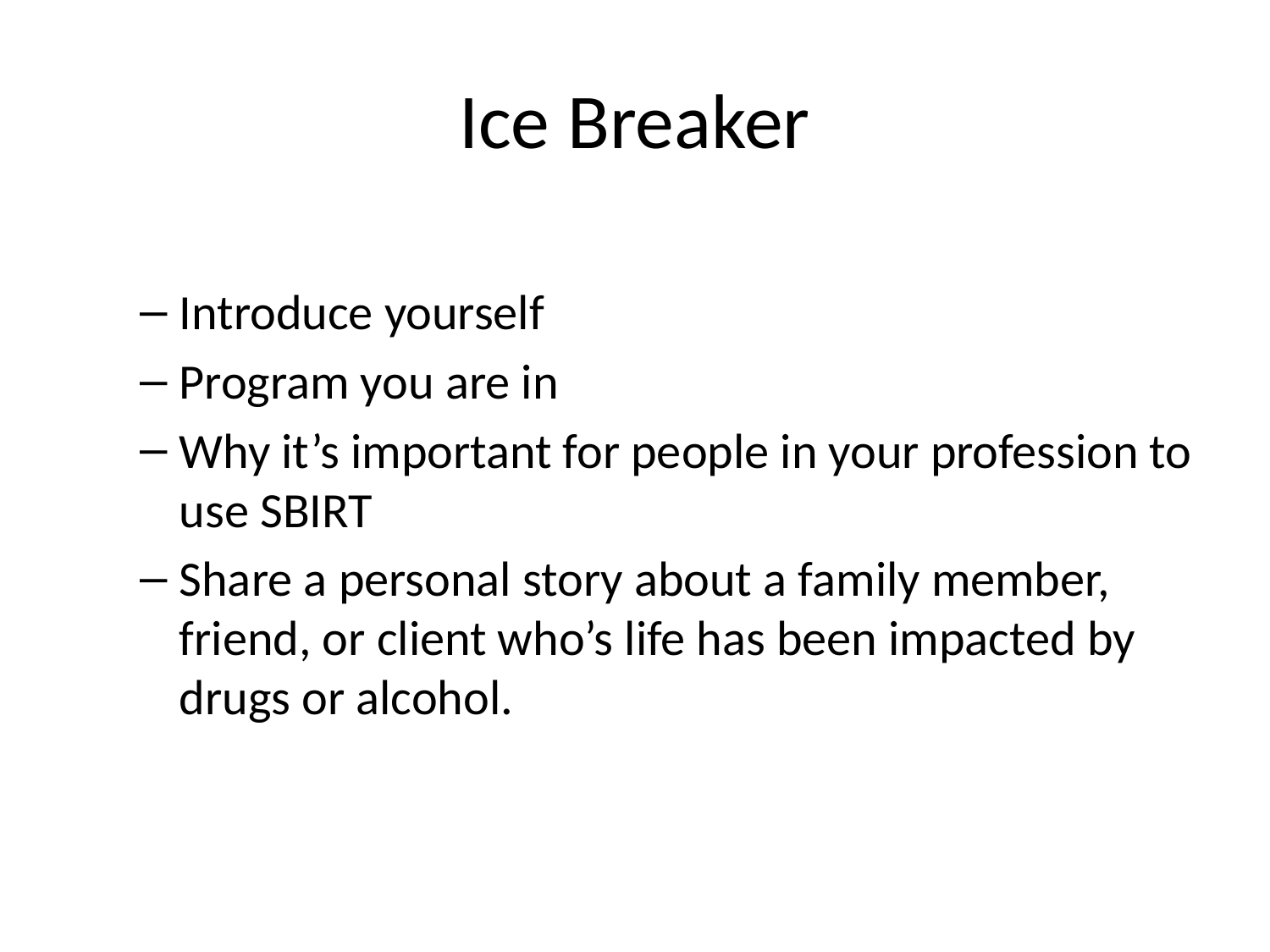

# Ice Breaker
Introduce yourself
Program you are in
Why it’s important for people in your profession to use SBIRT
Share a personal story about a family member, friend, or client who’s life has been impacted by drugs or alcohol.

## Slide 3
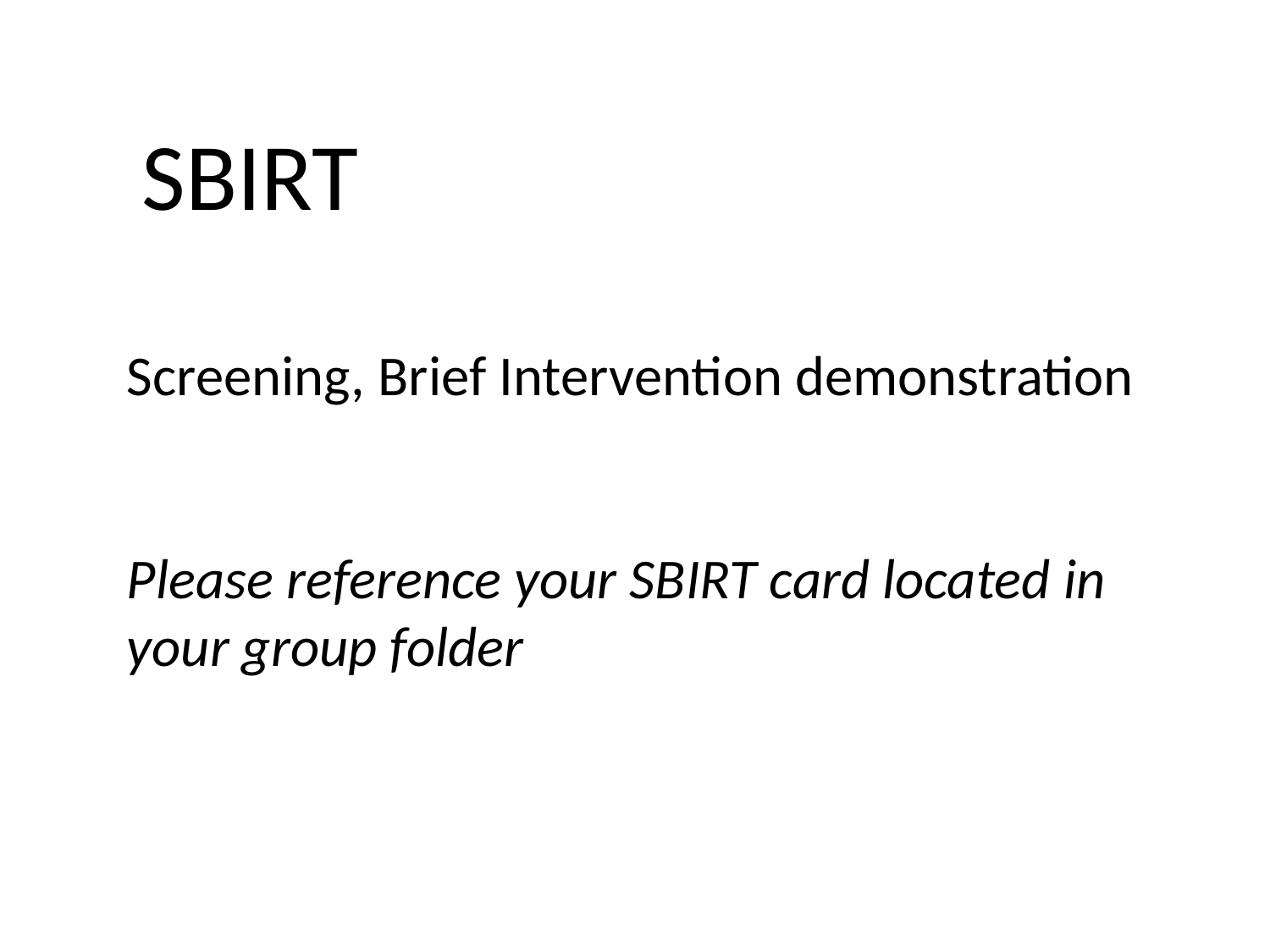

SBIRT
Screening, Brief Intervention demonstration
Please reference your SBIRT card located in your group folder

## Slide 4
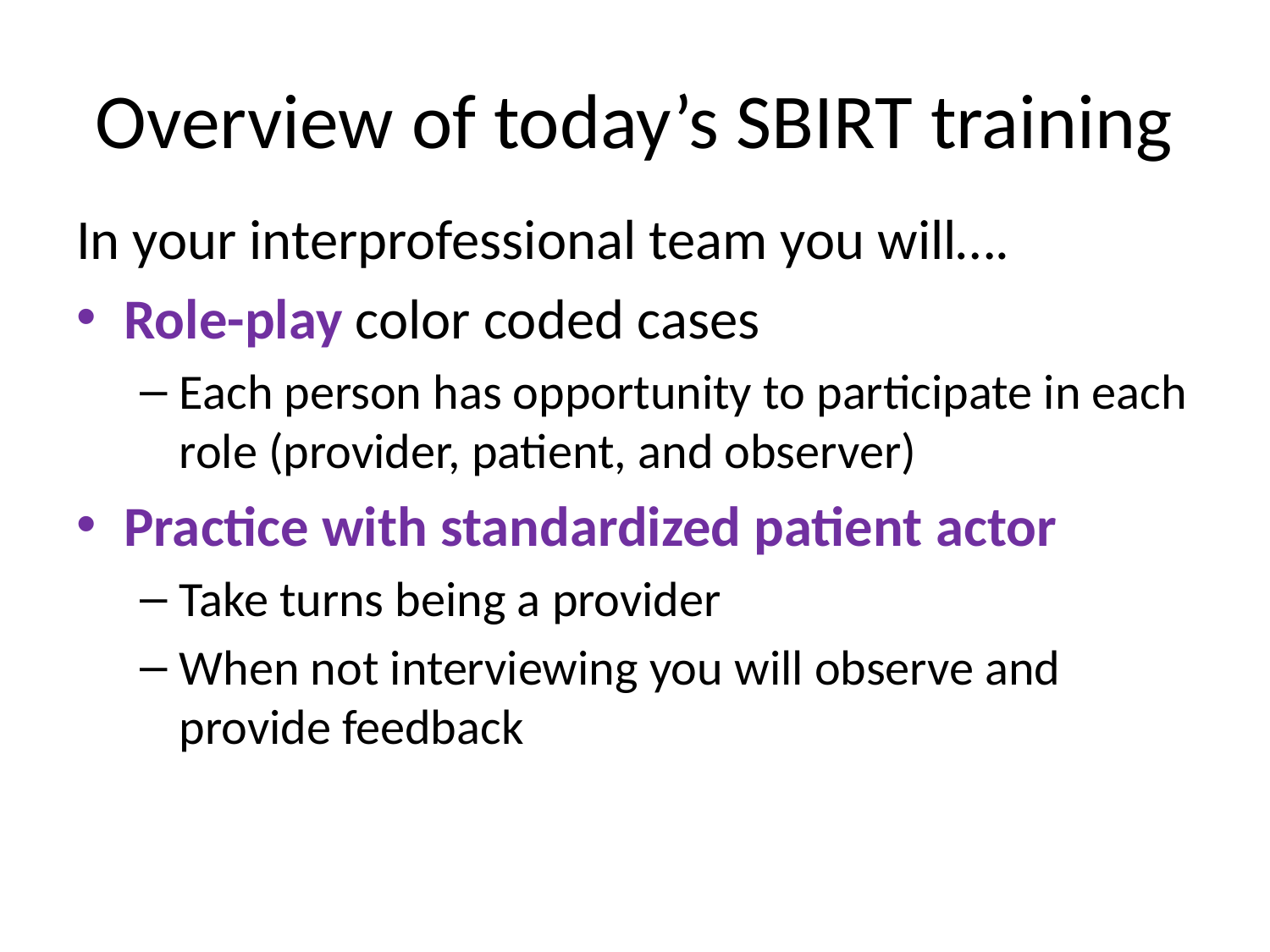

# Overview of today’s SBIRT training
In your interprofessional team you will….
Role-play color coded cases
Each person has opportunity to participate in each role (provider, patient, and observer)
Practice with standardized patient actor
Take turns being a provider
When not interviewing you will observe and provide feedback

## Slide 5
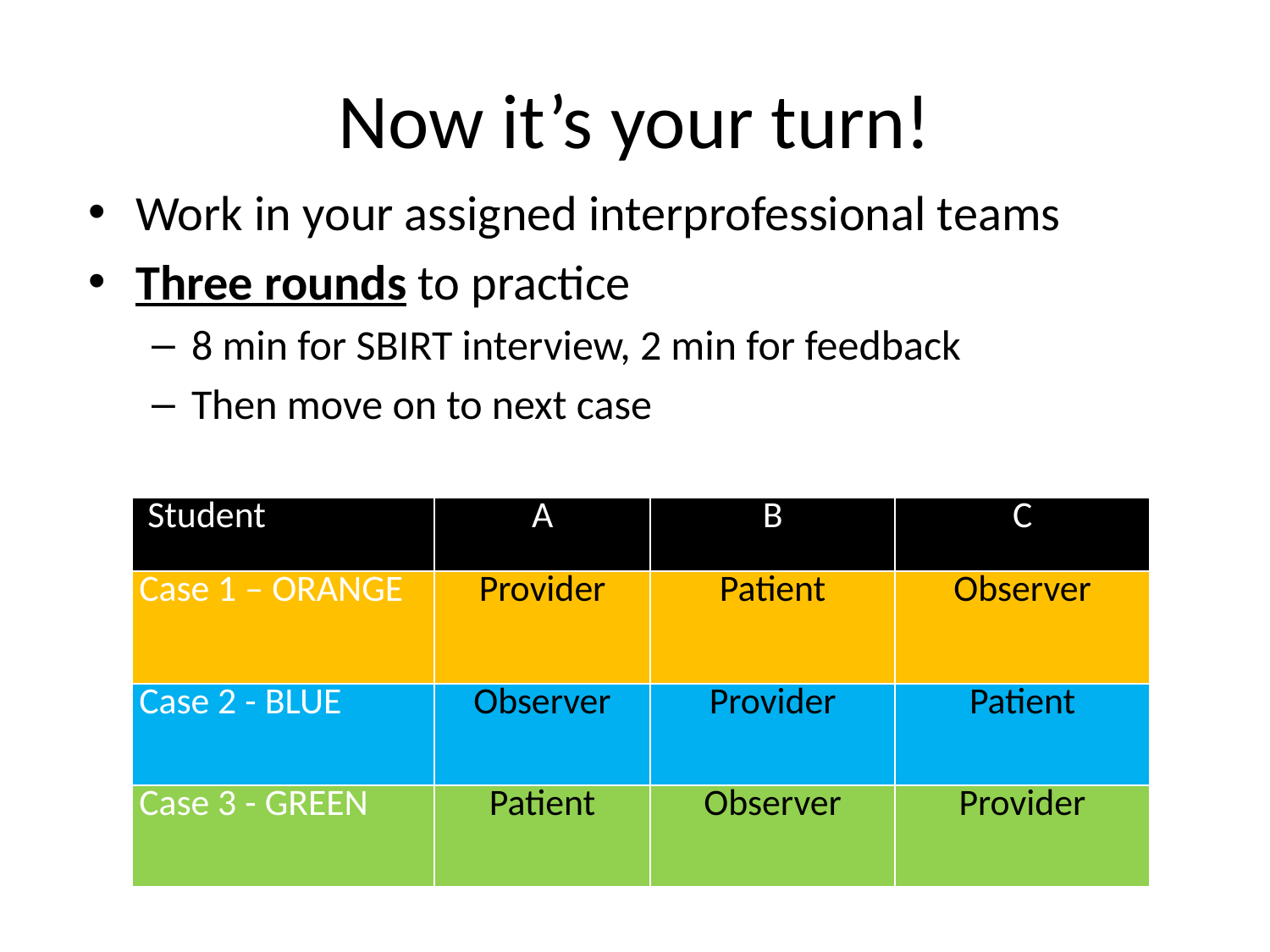

# Now it’s your turn!
Work in your assigned interprofessional teams
Three rounds to practice
8 min for SBIRT interview, 2 min for feedback
Then move on to next case
| Student | A | B | C |
| --- | --- | --- | --- |
| Case 1 – ORANGE | Provider | Patient | Observer |
| Case 2 - BLUE | Observer | Provider | Patient |
| Case 3 - GREEN | Patient | Observer | Provider |

## Slide 6
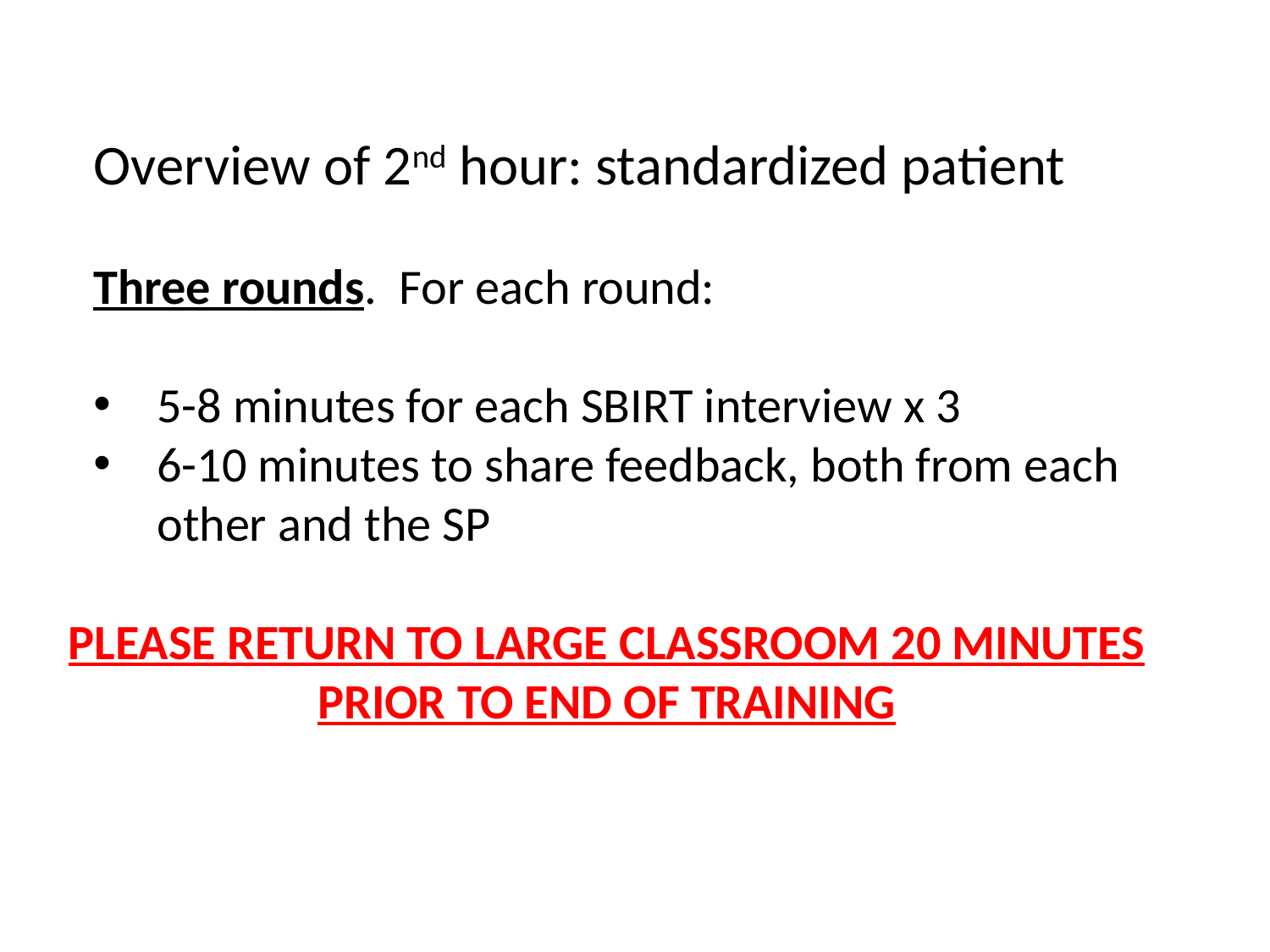

Overview of 2nd hour: standardized patient
Three rounds. For each round:
5-8 minutes for each SBIRT interview x 3
6-10 minutes to share feedback, both from each other and the SP
PLEASE RETURN TO LARGE CLASSROOM 20 MINUTES PRIOR TO END OF TRAINING

## Slide 7
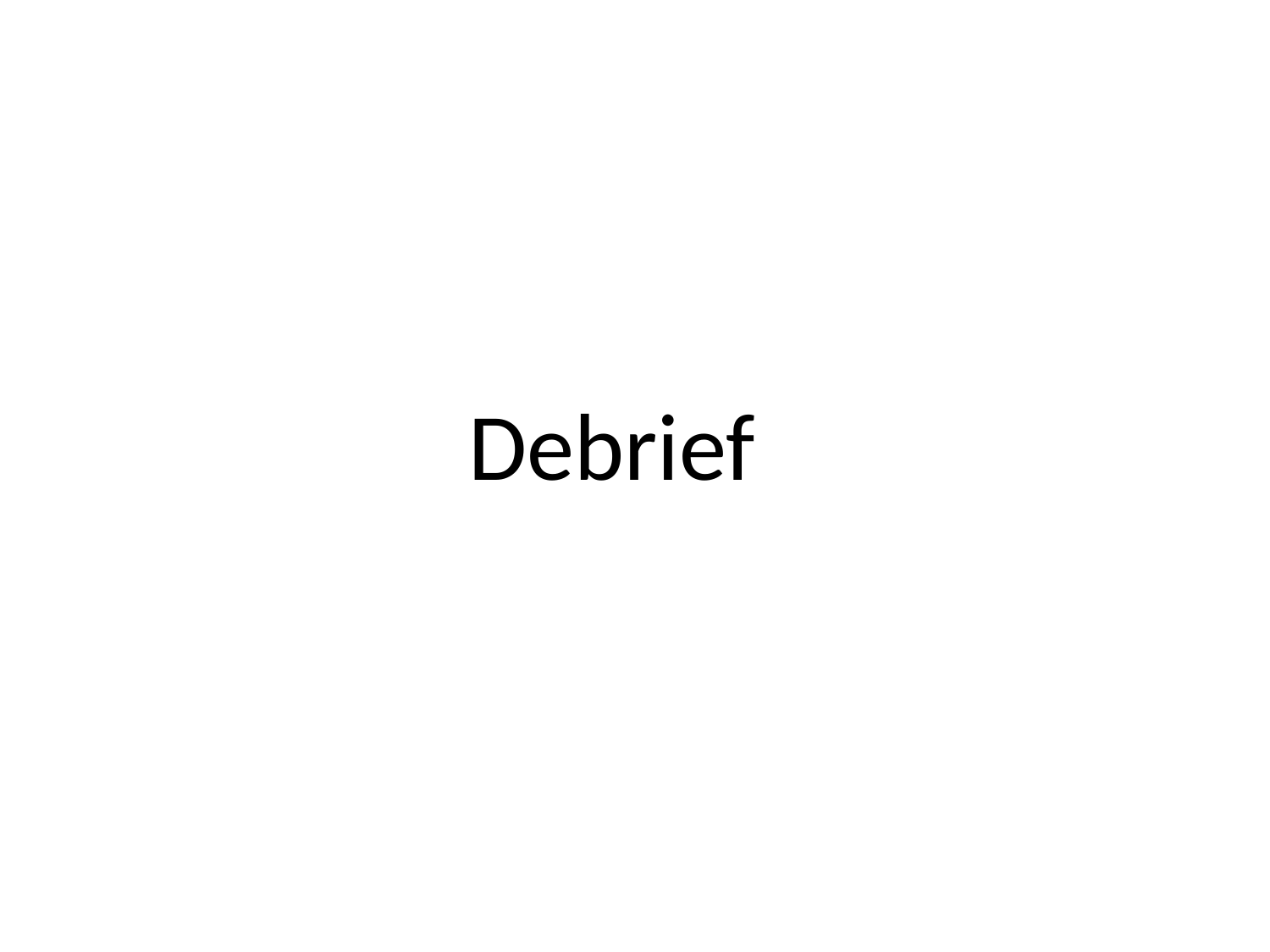

Debrief

## Slide 8
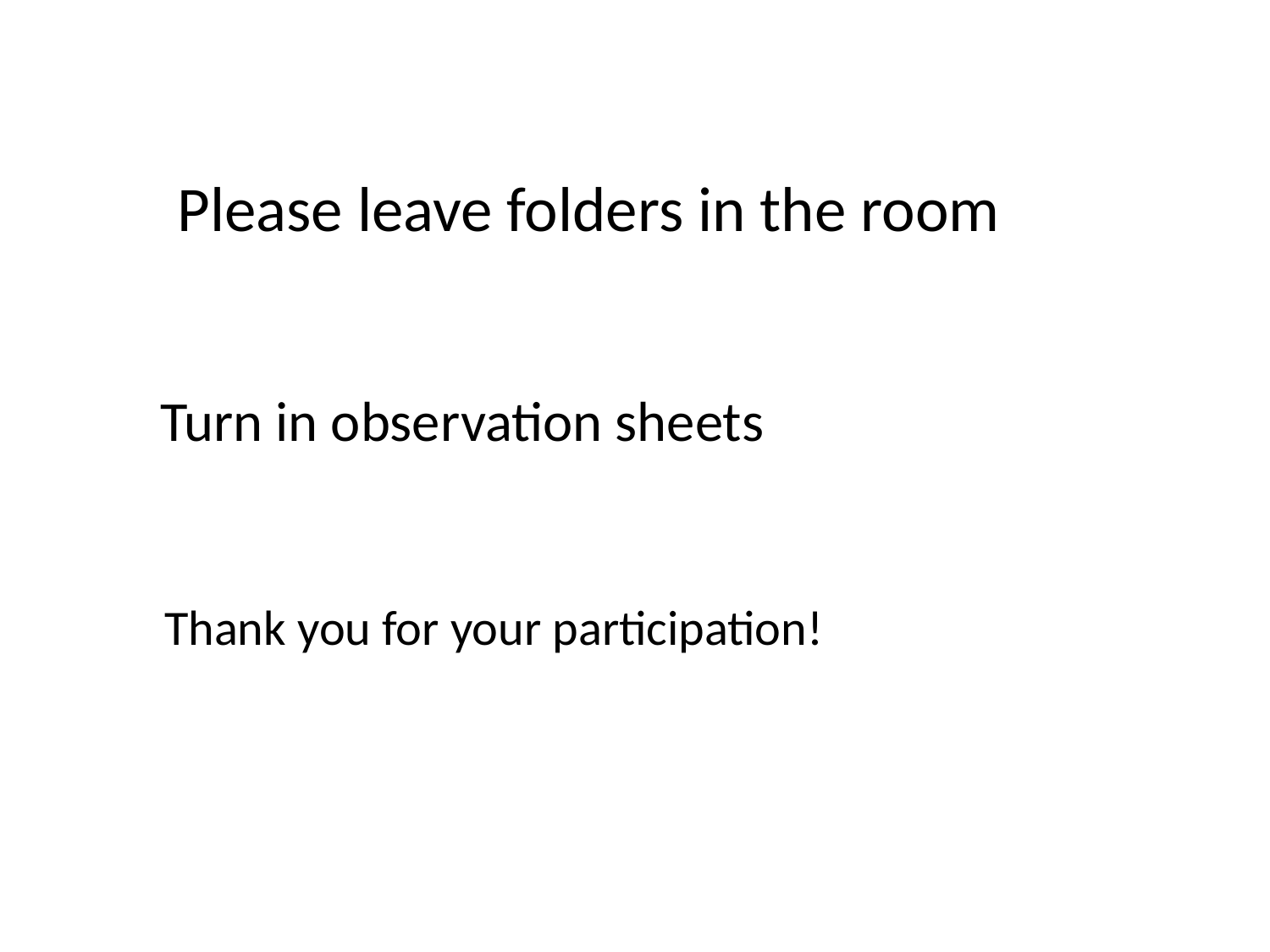

Please leave folders in the room
Turn in observation sheets
Thank you for your participation!
