# Supplementary material for: Standardized Patient Simulation Using SBIRT (Screening, Brief Intervention, and Referral for Treatment) as a Tool for Interprofessional Learning
Source: MedEdPORTAL. 2020 Sep 11;16:10955. doi: 10.15766/mep_2374-8265.10955 (PMC7485913; doi:10.15766/mep_2374-8265.10955)
Supplement: Supplementary file 1 — Educational Objectives.docxAdministrative Instructions Prior to Session.docxStudent Overview of SBIRT Components - Email Prior.docxStudent Prep - ADEPT Video.mp4AUDIT Screening Tool - Email and Print.docxDemonstration - SBIRT Colorado.mp4Faculty Overview and Agenda.docxSBIRT Slides for Live Session.pptxFaculty Script for Slide Presentation.docxSBIRT Pocket Card - Print.pdfStudent Agenda - Print.docxPeer Role-Play Case 1-Print ORANGE-Observer.docxPeer Role-Play Case 1-Print ORANGE-Patient.docxPeer Role-Play Case 1-Print ORANGE-Provider.docxPeer Role-Play Case 2-Print BLUE-Observer.docxPeer Role-Play Case 2-Print BLUE-Patient.docxPeer Role-Play Case 2-Print BLUE-Provider.docxPeer Role-Play Case 3-Print GREEN-Observer.docxPeer Role-Play Case 3-Print GREEN-Patient.docxPeer Role-Play Case 3-Print GREEN-Provider.docxSP Case Jamie Quimby.docxSP AUDIT Screen Jamie Quimby.pdfSP Case Pat Stewart.docxSP AUDIT Screen Pat Stewart.pdfEvaluation Tool.docx [file mep_2374-8265.10955-s001.zip › I. Faculty Script for Slide Presentation.docx]

**SBIRT Faculty Script**

**For use with *Appendix H (SBIRT slides for live session)***

**Slide 1**

Welcome to the Interprofessional SBIRT training session. My name is __________________________. Joining me as faculty for today’s session are ________________ from the College of ___________ and _______________________ from __________________________. Also here providing support is ____________, and our Standardized Patient Coordinator, _________.

SBIRT is a skill that can and should be implemented in a wide variety of settings by trained healthcare professionals. You’ve been assigned to an interprofessional team for today’s program, so you will have an opportunity to work with a student from at least one other health profession.

**Slide 2: Ice breaker**

*Ice breaker:*

*(*1) introduce yourself to your other two team members sharing, name, your program, and why you think it’s important for people in your profession to use SBIRT.

(2) Share a deidentified story about a client/patient, or friend or family member whose life has been impacted by use of drugs and / or alcohol. Please don’t use anyone’s name in order to protect your family, friend, or patient’s identity. Limit responses to 2 minutes per person.

**Slide 3: SBIRT**

In preparation for this session you watched videos demonstrating the process using motivational interviewing for Screening and Brief Intervention. Please pull out two items from your packets: the SBIRT pocket card and one of the colored sheets with the “OBSERVER” watermark, and use them as references. We are going to re-watch the demonstration video and pause to discuss tools we see the interviewer using.

**(*PLAY APPENDIX F video****, “Demonstration – SBIRT Colorado”*)

*[Pause video intermittently and review tools you see the interviewer using:*

- *raise the subject (“If it’s all right with you, I’d like to talk to you about your screening results”)*
- *normalization (“…something we ask everyone”)*
- *reflection (“I hear you saying…”)*
- *information sharing (“I’d like to share with you…”)*
- *provide feedback (“when drinking more than the low risk drinking limits, it can…”)*
- *ask-tell-ask (ask permission, share information, ask what patient thinks)*
- *ask patient for ideas (“what are some things you could do…”)*
- *enhance motivation (pros of change, readiness ruler)*
- *ask about pros and cons of change (“what are other reasons why lowering drinking might be important to you?”)*
- *readiness ruler (“why didn’t you choose a lower number?” to elicit change talk)*
- *elicit barriers and problem solve (“what might be some challenges…”)*
- *negotiate plan and advise (“how might you make these changes in your life?”)*
- *validation (“it sounds like you’ve come up with a great plan” and “it sounds like your health is really important to you”)*

Now this was a video of a patient who was very compliant. What would you do if you talked to a patient who was ambivalent or resistant to change? *Have students raise hands*.

*[Provide suggestions on how to respond to ambivalence….. (i.e. reflective listening, explore why the resistance is happening, summarizing, double-sided reflection: i.e. “I hear you say that you enjoy drinking and it’s a good release, and I also hear you say that alcohol drinking is affecting your marriage and your job responsibilities.” )]*

To close/end, respect the client’s right to self-determination and thank them for their time.

**Slide 4: Overview**

There are 2 main components: a role play to practice what you learned through pre-class module, then apply your knowledge with a standardized patient actor

**Slide 5 – “Now it’s your turn”**

Now it’s your turn to practice. Here’s how it will work. Each of you was given a folder when you arrived. On the front of the folder is your team number and the letters A, B, or C. We will have 3 rounds, each lasting 10 minutes. For each round, one person on your team will be the provider, one person will play the role of a patient, and the third person will serve as an observer that will give feedback to the provider (the person using SBIRT) at the conclusion of the interaction.

For the first round (case 1), use the orange colored sheets, for the second round (case 2) use the blue sheets, and the third round (case 3), the green sheets. If you are the observer, please fill out the color coded observation form that is provided in the folder. You don’t need to write anyone’s name on the observation sheet, but please turn in all of the observation sheets before you leave.

The brief negotiated interview should be just that – brief, lasting no longer than 8 minutes. I will ring this bell at the end of 8 minutes, so you know you have 2 remaining minutes to wrap up your interaction and receive feedback from the observer and patient. Do you have any questions?

*Note – sound a bell at 8 minutes reminding students to wrap up their conversations and allow the observer to give feedback to the provider. Sound a bell again 2 minutes later, and say, “*that’s the end of round 1. It’s time to switch roles and repeat the process. This time use the blue pages in your folders. “

*Then repeat the process, sound a bell at 8 minutes reminding students to wrap up their conversations so the observer has 2 minutes to provide feedback. Sound the bell two minutes later and say,* “that’s the end of round 2. It’s time to switch roles and repeat the process on last time. This time use the green pages in your folders. “

*Sound the bell at 8 minutes, and ask students to wrap up their dialogue and allow the observer to share feedback with the “provider.”*

*At the conclusion of the practice session, sound the bell for the last time.*

*Ask students to give you their full attention so you can explain the agenda for the 2^nd^ hour.*

**Slide 6: Second hour overview**

In the next 30 minutes, you will each have an opportunity to practice using SBIRT with a standardized patient, who is an actor that has been trained to interact with you about their drug or alcohol use.

Take a 5 minute break, then go with your team to your assigned room. When you get to your assigned room, your patient will already be there, much like a clinical setting. Each of you will take turns role playing an SBIRT interview with the standardized patient. Each of you will be given the same “provider” information and you will interview the SP consecutively. You will have only 5-8 minutes to interview the patient before switching. At the end of the 3 interviews, you will have 6-10 minutes to share feedback with each other and receive feedback from your SP.

Make sure someone assumes the role of time keeper each round, as there won’t be a faculty member there to time you. Please return to this room by ____________________ (20 minutes before end of session).

**Slide 7: Debrief**

Thank you for participating today. In the last few minutes we’d like to do a short debrief about the interprofessional collaborations you experienced today.

Sample debrief questions:

1. Why do you think *interprofessional* SBIRT training is important?
2. What parts of the SBIRT training session helped you have a better understanding of the roles or responsibilities of another health profession?
3. How will you use the knowledge and skills you gained through the SBIRT training in your practice?

*Wait for several student responses to each question before moving on to the next one. If asked a specific question, ask the group if anyone knows the answer before answering yourself.*

**Slide 8: final comments**

Thank you for your participation today. Be sure to leave your folders in the room when you leave. Also turn in any remaining observer forms.
