# Supplementary material for: Standardized Patient Simulation Using SBIRT (Screening, Brief Intervention, and Referral for Treatment) as a Tool for Interprofessional Learning
Source: MedEdPORTAL. 2020 Sep 11;16:10955. doi: 10.15766/mep_2374-8265.10955 (PMC7485913; doi:10.15766/mep_2374-8265.10955)
Supplement: Supplementary file 1 — Educational Objectives.docxAdministrative Instructions Prior to Session.docxStudent Overview of SBIRT Components - Email Prior.docxStudent Prep - ADEPT Video.mp4AUDIT Screening Tool - Email and Print.docxDemonstration - SBIRT Colorado.mp4Faculty Overview and Agenda.docxSBIRT Slides for Live Session.pptxFaculty Script for Slide Presentation.docxSBIRT Pocket Card - Print.pdfStudent Agenda - Print.docxPeer Role-Play Case 1-Print ORANGE-Observer.docxPeer Role-Play Case 1-Print ORANGE-Patient.docxPeer Role-Play Case 1-Print ORANGE-Provider.docxPeer Role-Play Case 2-Print BLUE-Observer.docxPeer Role-Play Case 2-Print BLUE-Patient.docxPeer Role-Play Case 2-Print BLUE-Provider.docxPeer Role-Play Case 3-Print GREEN-Observer.docxPeer Role-Play Case 3-Print GREEN-Patient.docxPeer Role-Play Case 3-Print GREEN-Provider.docxSP Case Jamie Quimby.docxSP AUDIT Screen Jamie Quimby.pdfSP Case Pat Stewart.docxSP AUDIT Screen Pat Stewart.pdfEvaluation Tool.docx [file mep_2374-8265.10955-s001.zip › J. SBIRT Pocket Card - Print.pdf]

Low-risk drinking limits

	Drinks per week	Drinks per day
Men	14	4
Women	7	3
All ages >65	7	3
Pregnancy	0	0

Categories of patient drinking

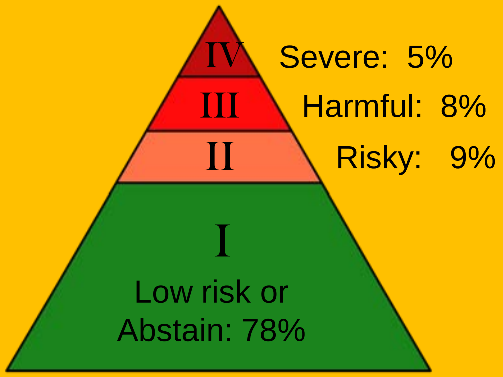

Readiness ruler:

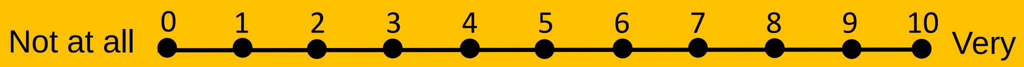

Raise the subject

- "Thank you for completing this questionnaire - is it ok if we review your results?"
- "Can you tell me more about your drinking or drug use? What does a typical week look like?"

Provide feedback

- "I recommend drinking below low-risk limits to help prevent new health problems or make existing ones worse."
- "Many patients who score into this zone have difficulty just cutting back and need to abstain from drinking alcohol."

Enhance motivation

- "What do you like and what are you concerned about when it comes to your alcohol/drug use?"
- "On a scale of 0-10, how ready are you to cut back/seek specialized treatment? Why not a lower number?"

Negotiate plan

- Summarize conversation. Then: "What steps do you think you can take to reach your goal of cutting back/seeking specialized treatment?"

Oregon alcohol & drug referral helpline:
1-800-923-4357

SBIRT
www.sbirtoregon.org
