# Supplementary material for: Standardized Patient Simulation Using SBIRT (Screening, Brief Intervention, and Referral for Treatment) as a Tool for Interprofessional Learning
Source: MedEdPORTAL. 2020 Sep 11;16:10955. doi: 10.15766/mep_2374-8265.10955 (PMC7485913; doi:10.15766/mep_2374-8265.10955)
Supplement: Supplementary file 1 — Educational Objectives.docxAdministrative Instructions Prior to Session.docxStudent Overview of SBIRT Components - Email Prior.docxStudent Prep - ADEPT Video.mp4AUDIT Screening Tool - Email and Print.docxDemonstration - SBIRT Colorado.mp4Faculty Overview and Agenda.docxSBIRT Slides for Live Session.pptxFaculty Script for Slide Presentation.docxSBIRT Pocket Card - Print.pdfStudent Agenda - Print.docxPeer Role-Play Case 1-Print ORANGE-Observer.docxPeer Role-Play Case 1-Print ORANGE-Patient.docxPeer Role-Play Case 1-Print ORANGE-Provider.docxPeer Role-Play Case 2-Print BLUE-Observer.docxPeer Role-Play Case 2-Print BLUE-Patient.docxPeer Role-Play Case 2-Print BLUE-Provider.docxPeer Role-Play Case 3-Print GREEN-Observer.docxPeer Role-Play Case 3-Print GREEN-Patient.docxPeer Role-Play Case 3-Print GREEN-Provider.docxSP Case Jamie Quimby.docxSP AUDIT Screen Jamie Quimby.pdfSP Case Pat Stewart.docxSP AUDIT Screen Pat Stewart.pdfEvaluation Tool.docx [file mep_2374-8265.10955-s001.zip › K. Student Agenda - Print.docx]

**Substance Abuse and Mental health Services Administration (SAMSHA) / Screening, Brief Intervention & referral for Treatment (SBIRT) Interprofessional Training Agenda**

Welcome and session overview (3 minutes)

Ice Breaker and Brief Review of SBIRT (22 minutes)

***Ice breaker:***

- (1) introduce yourself to your other two team members sharing, name, your program, and why you think it’s important for people in your profession to use SBIRT.
- (2) Share a deidentified story about a client/patient, or friend or family member whose life has been impacted by use of drugs and / or alcohol. *Please don’t use anyone’s name in order to protect confidentiality.*

Practice SBIRT in student triads (3 x 10 minutes = 30 minutes)

- **Three rounds** to practice
  - For each round: 8 minutes for SBIRT interview, then 2 minutes to provide constructive feedback to the “provider,” then switch to the next case

|  | **A** | **B** | **C** |
| --- | --- | --- | --- |
| **Case 1 – ORANGE** | Provider | Patient | Observer |
| **Case 2 - BLUE** | Observer | Provider | Patient |
| **Case 3 - GREEN** | Patient | Observer | Provider |

**If you’re having a difficult time starting the conversation, use one of these prompts:**

1. *Thank you for coming to our clinic today and filling out the questionnaire about your use of alcohol. I really appreciate your honest responses. Would you mind if we talked about your alcohol use?*
2. *It’s nice to see you again. Thank you for filling out the questionnaire about your use of alcohol. I’d like to*

*talk with you about your responses. Would that be okay?*

1. *I see that you’re here to talk about ______. I’d also like to talk with you about your alcohol use if that’s okay.*

Break and move to small rooms for practice interviews with standardized patients (5 minutes)

SBIRT interviews with standardized patients (3 x 10 minutes = 30 minutes)

- **Three rounds** to practice
  - For each round:
    - *Each student takes turns being provider; all 3 interviews done consecutively*
    - 8 min for each student to complete SBIRT interview (total 15-20 min for all 3)
    - After all 3 interviews, 6-10 min to share constructive feedback from one another and from the SP

Break and return to large group classroom (5 Minutes)

Course evaluation (10 minutes)

Large Group Debrief (10 minutes)

Adjourn

***Please leave folders in the room. Turn in observer forms. Thank you!***
