# Supplementary material for: Standardized Patient Simulation Using SBIRT (Screening, Brief Intervention, and Referral for Treatment) as a Tool for Interprofessional Learning
Source: MedEdPORTAL. 2020 Sep 11;16:10955. doi: 10.15766/mep_2374-8265.10955 (PMC7485913; doi:10.15766/mep_2374-8265.10955)
Supplement: Supplementary file 1 — Educational Objectives.docxAdministrative Instructions Prior to Session.docxStudent Overview of SBIRT Components - Email Prior.docxStudent Prep - ADEPT Video.mp4AUDIT Screening Tool - Email and Print.docxDemonstration - SBIRT Colorado.mp4Faculty Overview and Agenda.docxSBIRT Slides for Live Session.pptxFaculty Script for Slide Presentation.docxSBIRT Pocket Card - Print.pdfStudent Agenda - Print.docxPeer Role-Play Case 1-Print ORANGE-Observer.docxPeer Role-Play Case 1-Print ORANGE-Patient.docxPeer Role-Play Case 1-Print ORANGE-Provider.docxPeer Role-Play Case 2-Print BLUE-Observer.docxPeer Role-Play Case 2-Print BLUE-Patient.docxPeer Role-Play Case 2-Print BLUE-Provider.docxPeer Role-Play Case 3-Print GREEN-Observer.docxPeer Role-Play Case 3-Print GREEN-Patient.docxPeer Role-Play Case 3-Print GREEN-Provider.docxSP Case Jamie Quimby.docxSP AUDIT Screen Jamie Quimby.pdfSP Case Pat Stewart.docxSP AUDIT Screen Pat Stewart.pdfEvaluation Tool.docx [file mep_2374-8265.10955-s001.zip › L. Peer Role-Play Case 1-Print ORANGE-Observer.docx]

**Brief Intervention Observation Sheet**

***Did the Provider …***

| **(1)**  **Raise the subject** | **1)** Explain role and respectfully ask permission to have a discussion about alcohol/drug use | **Yes No** | **Comments** |  |  |  |
| --- | --- | --- | --- | --- | --- | --- |
|  | **2)** Review patient’s alcohol/drug use patterns | **Yes No** |  |  |  |  |
| **(2)**  **Provide feedback** | **3)** Share the patient’s AUDIT/DAST scores and zones | **Yes No** |  |  |  |  |
|  | **4)** Review low-risk guidelines relevant to his/her sex and age group | **Yes No** |  |  |  |  |
|  | **5)** Explore possible connection to health, social, work issues and express concern(s) (if relevant) | **Yes No** |  |  |  |  |
| **(3)**  **Enhance motivation** | **6)** Ask patient to select a number on the “Readiness Ruler” | **Yes No** |  |  |  |  |
|  | **6a)** What was the number? |  |  |  |  |  |
|  | **7)** Ask patient: *why didn’t you pick a lower number?* OR Ask patient: *how would your drinking (drug use) have to impact your life in order for you to start thinking about cutting back*? OR Discuss patient’s pros and cons of use | **Yes No** |  |  |  |  |
| **(4) Negotiate a plan** | **8)** Provide a summary of readiness (You said ...) | **Yes No** |  |  |  |  |
|  | **9)** Negotiate a goal with the patient based on his/her response to: *What steps would you be willing to take?* | **Yes No** |  |  |  |  |
|  | **10)** Offer a menu of choices for change, provide recommendation, secure agreement | **Yes No** |  |  |  |  |
| **Motivation** | **11)** To what degree did the provider use a motivational style (open-ended questions, reflective listening, not confrontational)?   \| ***Not At All*** \|  \|  \|  \|  \|  \| ***Very Effectively*** \| \| --- \| --- \| --- \| --- \| --- \| --- \| --- \| \| **1** \| **2** \| **3** \| **4** \| **5** \| **6** \| **7** \| \|  \|  \|  \|  \|  \|  \|  \| |  |  |  |  |  |

Adapted from the BI Adherence/Competence Scale: Pantalon MV, Martino S, Dziura J, et al. (2012). Development of a scale to measure practitioner adherence to a brief intervention in the emergency department. *J Subst Abuse Treat*. 2012;43(4):382–388. doi:10.1016/j.jsat.2012.08.011
