# Supplementary material for: Standardized Patient Simulation Using SBIRT (Screening, Brief Intervention, and Referral for Treatment) as a Tool for Interprofessional Learning
Source: MedEdPORTAL. 2020 Sep 11;16:10955. doi: 10.15766/mep_2374-8265.10955 (PMC7485913; doi:10.15766/mep_2374-8265.10955)
Supplement: Supplementary file 1 — Educational Objectives.docxAdministrative Instructions Prior to Session.docxStudent Overview of SBIRT Components - Email Prior.docxStudent Prep - ADEPT Video.mp4AUDIT Screening Tool - Email and Print.docxDemonstration - SBIRT Colorado.mp4Faculty Overview and Agenda.docxSBIRT Slides for Live Session.pptxFaculty Script for Slide Presentation.docxSBIRT Pocket Card - Print.pdfStudent Agenda - Print.docxPeer Role-Play Case 1-Print ORANGE-Observer.docxPeer Role-Play Case 1-Print ORANGE-Patient.docxPeer Role-Play Case 1-Print ORANGE-Provider.docxPeer Role-Play Case 2-Print BLUE-Observer.docxPeer Role-Play Case 2-Print BLUE-Patient.docxPeer Role-Play Case 2-Print BLUE-Provider.docxPeer Role-Play Case 3-Print GREEN-Observer.docxPeer Role-Play Case 3-Print GREEN-Patient.docxPeer Role-Play Case 3-Print GREEN-Provider.docxSP Case Jamie Quimby.docxSP AUDIT Screen Jamie Quimby.pdfSP Case Pat Stewart.docxSP AUDIT Screen Pat Stewart.pdfEvaluation Tool.docx [file mep_2374-8265.10955-s001.zip › M. Peer Role-Play Case 1-Print ORANGE-Patient.docx]

**Orange/Yellow: Role Play Case 1: Sophie**

**PATIENT (For the patient to read):**

You are a 20-year old college student (DOB: January 3, 1996) who was in a car accident early Saturday morning as you swerved into the median “to avoid a dog crossing the road”. Or, at least that’s the story you told the police officer who responded as well as the tow truck driver and eventually your parents too. When they asked why you were out so late, you stated that you were at the library studying until midnight when it closes. You came to the ER with concerns over pain in your neck, weakness in your upper extremities and a bad headache. Your symptoms are interfering with your activities of daily living (ADLs).

However you omitted that you then stopped by a local nightclub where you were able to access the patio entrance without showing your fake ID. *It is okay to share it with the practitioner if you are asked though.*

- You will sometimes get out with friends but are only three months away from 21 and worry about getting caught.
- You do go out once or twice a week, you usually only drink until you are buzzed, maybe 4 or 5 drinks (depends on who is buying).
- You did not initially want to go to the hospital earlier because you were worried they would do an alcohol test, and you were trying to make sure the officer was not too suspicious.
- At this point, you don't think you have a drinking problem, although your friends have expressed concerns about your increased drinking and passing out several times lately. In addition, two of your professors have expressed concern over your tardiness issue and its impact on your grades. You are anxious about maintaining a 3.2 or higher GPA, which is required for your scholarship.
- At the ER, you to complete an AUDIT alcohol screening questionnaire upon request from one of the nurses
- Your level of readiness for change is a 5.

**ALCOHOL USE QUESTIONS (AUDIT)**

Drinking alcohol can affect your health and some medications you may take. Please help us provide you with the best medical care by answering the questions below.

| **QUESTIONS** | **0** | **1** | **2** | **3** | **4** | **5** | **6** | **Score** |
| --- | --- | --- | --- | --- | --- | --- | --- | --- |
| 1. How often do you have a drink containing alcohol? | Never | Less than monthly | Monthly | Weekly | 2-3 times a week | 4-6 times a week | Daily | **2** |
| 2. How many drinks containing alcohol do you have on a typical day you are drinking? | 1 drink | 2 drinks | 3 drinks | 4 drinks | 5-6 drinks | 7-8  drinks | 10 or more drinks | **4** |
| 3. How often do you have X (5 for men; 4 for women & men over age 65) or more drinks on one occasion? | Never | Less than monthly | Monthly | Weekly | 2-3 times a week | 4-6 times a week | Daily | **3** |
| 4. How often during the last year have you found that you were not able to stop drinking once you had started? | Never | Less than monthly | Monthly | Weekly | Daily or almost daily |  |  | **0** |
| 5. How often during the past year have you failed to do what was expected of you because of drinking? | Never | Less than monthly | Monthly | Weekly | Daily or almost daily |  |  | **2** |
| 6. How often during the past year have you needed a drink first thing in the morning to get yourself going after a heavy drinking session? | Never | Less than monthly | Monthly | Weekly | Daily or almost daily |  |  | **0** |
| 7. How often during the past year have you had a feeling of guilt or remorse after drinking? | Never | Less than monthly | Monthly | Weekly | Daily or almost daily |  |  | **0** |
| 8. How often during the past year have you been unable to remember what happened the night before because you had been drinking? | Never | Less than monthly | Monthly | Weekly | Daily or almost daily |  |  | **0** |
| 9. Have you or someone else been injured because of your drinking? | No |  | Yes, but not in the past year |  | Yes, during the past year |  |  | **4** |
| 10. Has a relative, friend, doctor, or other health care worker been concerned about your drinking and suggested you cut down? | No |  | Yes, but not in the past year |  | Yes, during the past year |  |  | **4** |
|  | | | | | | | **Total *19*** | |
