# Supplementary material for: Standardized Patient Simulation Using SBIRT (Screening, Brief Intervention, and Referral for Treatment) as a Tool for Interprofessional Learning
Source: MedEdPORTAL. 2020 Sep 11;16:10955. doi: 10.15766/mep_2374-8265.10955 (PMC7485913; doi:10.15766/mep_2374-8265.10955)
Supplement: Supplementary file 1 — Educational Objectives.docxAdministrative Instructions Prior to Session.docxStudent Overview of SBIRT Components - Email Prior.docxStudent Prep - ADEPT Video.mp4AUDIT Screening Tool - Email and Print.docxDemonstration - SBIRT Colorado.mp4Faculty Overview and Agenda.docxSBIRT Slides for Live Session.pptxFaculty Script for Slide Presentation.docxSBIRT Pocket Card - Print.pdfStudent Agenda - Print.docxPeer Role-Play Case 1-Print ORANGE-Observer.docxPeer Role-Play Case 1-Print ORANGE-Patient.docxPeer Role-Play Case 1-Print ORANGE-Provider.docxPeer Role-Play Case 2-Print BLUE-Observer.docxPeer Role-Play Case 2-Print BLUE-Patient.docxPeer Role-Play Case 2-Print BLUE-Provider.docxPeer Role-Play Case 3-Print GREEN-Observer.docxPeer Role-Play Case 3-Print GREEN-Patient.docxPeer Role-Play Case 3-Print GREEN-Provider.docxSP Case Jamie Quimby.docxSP AUDIT Screen Jamie Quimby.pdfSP Case Pat Stewart.docxSP AUDIT Screen Pat Stewart.pdfEvaluation Tool.docx [file mep_2374-8265.10955-s001.zip › P. Peer Role-Play Case 2-Print BLUE-Patient.docx]

**Blue- Role Play Case 2: Steve**

**PATIENT (For the patient to read):**

Steve is a 27-year old male (DOB: 2-2-1989) presenting at a follow-up visit. He was recently prescribed antidepressants at a previous visit and he says he is still having some problems with “feeling down” sometimes. He also has complaints of numbness of his right forearm and right wrist weakness (he is right handed).

He is consuming about 3 drinks (beer or wine) 1-2 times on week days and he consumes 8-10 (beers) once or twice on the weekends.

- These are some of Steve’s thoughts and feelings about his drinking - he may or may not disclose depending on how he is approached by the doctor:
  - You are married and you drink during the week at home with your wife and on the weekends you go out with friends.
  - You’re not really worried about your drinking during the weekdays, but you know you over-due it at times on the weekends. In fact just this last month you had such a hang-over after being with your friends drinking on Sunday night that you had to call in sick. Your wife was not happy.
  - And there was that one time that you really couldn’t remember how you and your friends ended up at “Sully’s Bowling Alley.”
  - Your father is an alcoholic and you don’t want to end up like him.
  - There have been periods in your life that you decided to completely stop drinking and you were able to do that 2-3 months at a time.
  - You’re level of readiness for change is a 7.
- As part of your visit today, you complete an Alcohol Screening Questionnaire (AUDIT)

**ALCOHOL USE QUESTIONS (AUDIT)**

| **QUESTIONS** | **0** | **1** | **2** | **3** | **4** | **5** | **6** | **Score** |
| --- | --- | --- | --- | --- | --- | --- | --- | --- |
| 1. How often do you have a drink containing alcohol? | Never | Less than monthly | Monthly | Weekly | 2-3 times a week | 4-6 times a week | Daily | **4** |
| 2. How many drinks containing alcohol do you have on a typical day you are drinking? | 1 drink | 2 drinks | 3 drinks | 4 drinks | 5-6 drinks | 7-8  drinks | 10 or more drinks | **2** |
| 3. How often do you have X (5 for men; 4 for women & men over age 65) or more drinks on one occasion? | Never | Less than monthly | Monthly | Weekly | 2-3 times a week | 4-6 times a week | Daily | **3** |
| 4. How often during the last year have you found that you were not able to stop drinking once you had started? | Never | Less than monthly | Monthly | Weekly | Daily or almost daily |  |  | **0** |
| 5. How often during the past year have you failed to do what was expected of you because of drinking? | Never | Less than monthly | Monthly | Weekly | Daily or almost daily |  |  | **1** |
| 6. How often during the past year have you needed a drink first thing in the morning to get yourself going after a heavy drinking session? | Never | Less than monthly | Monthly | Weekly | Daily or almost daily |  |  | **0** |
| 7. How often during the past year have you had a feeling of guilt or remorse after drinking? | Never | Less than monthly | Monthly | Weekly | Daily or almost daily |  |  | **1** |
| 8. How often during the past year have you been unable to remember what happened the night before because you had been drinking? | Never | Less than monthly | Monthly | Weekly | Daily or almost daily |  |  | **1** |
| 9. Have you or someone else been injured because of your drinking? | No |  | Yes, but not in the past year |  | Yes, during the past year |  |  | **0** |
| 10. Has a relative, friend, doctor, or other health care worker been concerned about your drinking and suggested you cut down? | No |  | Yes, but not in the past year |  | Yes, during the past year |  |  | **4** |
|  | | | | | | | **Total *16*** | |

Drinking alcohol can affect your health and some medications you may take. Please help us provide you with the best medical care by answering the questions belo
