# Supplementary material for: Standardized Patient Simulation Using SBIRT (Screening, Brief Intervention, and Referral for Treatment) as a Tool for Interprofessional Learning
Source: MedEdPORTAL. 2020 Sep 11;16:10955. doi: 10.15766/mep_2374-8265.10955 (PMC7485913; doi:10.15766/mep_2374-8265.10955)
Supplement: Supplementary file 1 — Educational Objectives.docxAdministrative Instructions Prior to Session.docxStudent Overview of SBIRT Components - Email Prior.docxStudent Prep - ADEPT Video.mp4AUDIT Screening Tool - Email and Print.docxDemonstration - SBIRT Colorado.mp4Faculty Overview and Agenda.docxSBIRT Slides for Live Session.pptxFaculty Script for Slide Presentation.docxSBIRT Pocket Card - Print.pdfStudent Agenda - Print.docxPeer Role-Play Case 1-Print ORANGE-Observer.docxPeer Role-Play Case 1-Print ORANGE-Patient.docxPeer Role-Play Case 1-Print ORANGE-Provider.docxPeer Role-Play Case 2-Print BLUE-Observer.docxPeer Role-Play Case 2-Print BLUE-Patient.docxPeer Role-Play Case 2-Print BLUE-Provider.docxPeer Role-Play Case 3-Print GREEN-Observer.docxPeer Role-Play Case 3-Print GREEN-Patient.docxPeer Role-Play Case 3-Print GREEN-Provider.docxSP Case Jamie Quimby.docxSP AUDIT Screen Jamie Quimby.pdfSP Case Pat Stewart.docxSP AUDIT Screen Pat Stewart.pdfEvaluation Tool.docx [file mep_2374-8265.10955-s001.zip › Q. Peer Role-Play Case 2-Print BLUE-Provider.docx]

**Blue- Role Play Case 2: Steve**

**PROVIDER (For the clinician to read):**

Steve is a 27-year old male presenting at a follow-up visit. He was recently prescribed antidepressants at a previous visit and he says he is still having some problems with “feeling down” sometimes. He also has complaints of numbness of his right forearm and right wrist weakness.

**ALCOHOL USE QUESTIONS (AUDIT) SCORING:**

Each response from the AUDIT has a score ranging from 0 to 4. The top of each column has a number. That number equals the score value for responses in that column. After a patient has completed the AUDIT, add up each column score, and then sum all five columns for the patient’s score. Below are the scoring guidelines for the AUDIT.

| **Guidelines for Interpretation for AUDIT** | | |
| --- | --- | --- |
| **Score** | **Risk Level** | **Intervention** |
| 0-6 (*Female*)  0-7 (*Male*) | Zone I | Feedback and alcohol education |
| 7-15 (*Female*)  8-15 (*Male*) | Zone II | Brief intervention |
| 16-19 | Zone III | Brief intervention plus brief therapy |
| 20-40 | Zone IV | Brief intervention plus referral to chemical dependency treatment |

**ALCOHOL USE QUESTIONS (AUDIT)**

| **QUESTIONS** | **0** | **1** | **2** | **3** | **4** | **5** | **6** | **Score** |
| --- | --- | --- | --- | --- | --- | --- | --- | --- |
| 1. How often do you have a drink containing alcohol? | Never | Less than monthly | Monthly | Weekly | 2-3 times a week | 4-6 times a week | Daily | **4** |
| 2. How many drinks containing alcohol do you have on a typical day you are drinking? | 1 drink | 2 drinks | 3 drinks | 4 drinks | 5-6 drinks | 7-8  drinks | 10 or more drinks | **2** |
| 3. How often do you have X (5 for men; 4 for women & men over age 65) or more drinks on one occasion? | Never | Less than monthly | Monthly | Weekly | 2-3 times a week | 4-6 times a week | Daily | **3** |
| 4. How often during the last year have you found that you were not able to stop drinking once you had started? | Never | Less than monthly | Monthly | Weekly | Daily or almost daily |  |  | **0** |
| 5. How often during the past year have you failed to do what was expected of you because of drinking? | Never | Less than monthly | Monthly | Weekly | Daily or almost daily |  |  | **1** |
| 6. How often during the past year have you needed a drink first thing in the morning to get yourself going after a heavy drinking session? | Never | Less than monthly | Monthly | Weekly | Daily or almost daily |  |  | **0** |
| 7. How often during the past year have you had a feeling of guilt or remorse after drinking? | Never | Less than monthly | Monthly | Weekly | Daily or almost daily |  |  | **1** |
| 8. How often during the past year have you been unable to remember what happened the night before because you had been drinking? | Never | Less than monthly | Monthly | Weekly | Daily or almost daily |  |  | **1** |
| 9. Have you or someone else been injured because of your drinking? | No |  | Yes, but not in the past year |  | Yes, during the past year |  |  | **0** |
| 10. Has a relative, friend, doctor, or other health care worker been concerned about your drinking and suggested you cut down? | No |  | Yes, but not in the past year |  | Yes, during the past year |  |  | **4** |
|  | | | | | | | **Total *16*** | |

Drinking alcohol can affect your health and some medications you may take. Please help us provide you with the best medical care by answering the questions below.
