# Supplementary material for: Standardized Patient Simulation Using SBIRT (Screening, Brief Intervention, and Referral for Treatment) as a Tool for Interprofessional Learning
Source: MedEdPORTAL. 2020 Sep 11;16:10955. doi: 10.15766/mep_2374-8265.10955 (PMC7485913; doi:10.15766/mep_2374-8265.10955)
Supplement: Supplementary file 1 — Educational Objectives.docxAdministrative Instructions Prior to Session.docxStudent Overview of SBIRT Components - Email Prior.docxStudent Prep - ADEPT Video.mp4AUDIT Screening Tool - Email and Print.docxDemonstration - SBIRT Colorado.mp4Faculty Overview and Agenda.docxSBIRT Slides for Live Session.pptxFaculty Script for Slide Presentation.docxSBIRT Pocket Card - Print.pdfStudent Agenda - Print.docxPeer Role-Play Case 1-Print ORANGE-Observer.docxPeer Role-Play Case 1-Print ORANGE-Patient.docxPeer Role-Play Case 1-Print ORANGE-Provider.docxPeer Role-Play Case 2-Print BLUE-Observer.docxPeer Role-Play Case 2-Print BLUE-Patient.docxPeer Role-Play Case 2-Print BLUE-Provider.docxPeer Role-Play Case 3-Print GREEN-Observer.docxPeer Role-Play Case 3-Print GREEN-Patient.docxPeer Role-Play Case 3-Print GREEN-Provider.docxSP Case Jamie Quimby.docxSP AUDIT Screen Jamie Quimby.pdfSP Case Pat Stewart.docxSP AUDIT Screen Pat Stewart.pdfEvaluation Tool.docx [file mep_2374-8265.10955-s001.zip › S. Peer Role-Play Case 3-Print GREEN-Patient.docx]

**Green- Role Play Case 3: Joseph (or Josephine)**

**PATIENT (For the patient to read):**

You are a 35-year-old businessman/woman (DOB 2-4-1981). You have been very stressed out at work the past month. Normally you travel 3-4 nights per week, but this has increased lately, and you have had lots of deadlines. You are forced to eat out with business associates for lunch and dinner almost 3-4 days a week. You came to the ED today out of necessity. You have had increasing pain in your upper abdomen and constant nausea, which is affecting your ability to work and sleep. You have been taking Tums and Zantac over-the-counter sporadically, with brief periods of relief.

- Lately you have been drinking 1-2 drinks with lunch and 2-4 drinks with dinner.
- You drink scotch with a little water.
- The amount seems to have increased after your spouse brought up a separation/divorce and requested custody of the children
- You are initially ambivalent about changing your drinking behavior.
- Reasons for drinking:
  - All your associates have alcohol with meals and you do not want to stand out.
  - Also, alcohol makes you relax with clients, and you think it gives you an edge with negotiating.
- Reasons for considering changing alcohol use:
  - Your nausea and abdominal pain.
  - You are also having more trouble than usual sleeping in hotel rooms.
  - It is beginning to interfere with your ability to work

These are significant factors that once you realize they are directly related to drinking you are ready to take advice.

- You complete an Alcohol Screening Questionnaire (AUDIT) as part of your visit today
- Your level of readiness for change is a 5.

**ALCOHOL USE QUESTIONS (AUDIT)**

Drinking alcohol can affect your health and some medications you may take. Please help us provide you with the best medical care by answering the questions below

| **QUESTIONS** | **0** | **1** | **2** | **3** | **4** | **5** | **6** | **Score** |
| --- | --- | --- | --- | --- | --- | --- | --- | --- |
| 1. How often do you have a drink containing alcohol? | Never | Less than monthly | Monthly | Weekly | 2-3 times a week | 4-6 times a week | Daily | **5** |
| 2. How many drinks containing alcohol do you have on a typical day you are drinking? | 1 drink | 2 drinks | 3 drinks | 4 drinks | 5-6 drinks | 7-8  drinks | 10 or more drinks | **4** |
| 3. How often do you have X (5 for men; 4 for women & men over age 65) or more drinks on one occasion? | Never | Less than monthly | Monthly | Weekly | 2-3 times a week | 4-6 times a week | Daily | **6** |
| 4. How often during the last year have you found that you were not able to stop drinking once you had started? | Never | Less than monthly | Monthly | Weekly | Daily or almost daily |  |  | **0** |
| 5. How often during the past year have you failed to do what was expected of you because of drinking? | Never | Less than monthly | Monthly | Weekly | Daily or almost daily |  |  | **2** |
| 6. How often during the past year have you needed a drink first thing in the morning to get yourself going after a heavy drinking session? | Never | Less than monthly | Monthly | Weekly | Daily or almost daily |  |  | **0** |
| 7. How often during the past year have you had a feeling of guilt or remorse after drinking? | Never | Less than monthly | Monthly | Weekly | Daily or almost daily |  |  | **0** |
| 8. How often during the past year have you been unable to remember what happened the night before because you had been drinking? | Never | Less than monthly | Monthly | Weekly | Daily or almost daily |  |  | **0** |
| 9. Have you or someone else been injured because of your drinking? | No |  | Yes, but not in the past year |  | Yes, during the past year |  |  | **0** |
| 10. Has a relative, friend, doctor, or other health care worker been concerned about your drinking and suggested you cut down? | No |  | Yes, but not in the past year |  | Yes, during the past year |  |  | **0** |
|  | | | | | | | **Total *16*** | |
