# Supplementary material for: Standardized Patient Simulation Using SBIRT (Screening, Brief Intervention, and Referral for Treatment) as a Tool for Interprofessional Learning
Source: MedEdPORTAL. 2020 Sep 11;16:10955. doi: 10.15766/mep_2374-8265.10955 (PMC7485913; doi:10.15766/mep_2374-8265.10955)
Supplement: Supplementary file 1 — Educational Objectives.docxAdministrative Instructions Prior to Session.docxStudent Overview of SBIRT Components - Email Prior.docxStudent Prep - ADEPT Video.mp4AUDIT Screening Tool - Email and Print.docxDemonstration - SBIRT Colorado.mp4Faculty Overview and Agenda.docxSBIRT Slides for Live Session.pptxFaculty Script for Slide Presentation.docxSBIRT Pocket Card - Print.pdfStudent Agenda - Print.docxPeer Role-Play Case 1-Print ORANGE-Observer.docxPeer Role-Play Case 1-Print ORANGE-Patient.docxPeer Role-Play Case 1-Print ORANGE-Provider.docxPeer Role-Play Case 2-Print BLUE-Observer.docxPeer Role-Play Case 2-Print BLUE-Patient.docxPeer Role-Play Case 2-Print BLUE-Provider.docxPeer Role-Play Case 3-Print GREEN-Observer.docxPeer Role-Play Case 3-Print GREEN-Patient.docxPeer Role-Play Case 3-Print GREEN-Provider.docxSP Case Jamie Quimby.docxSP AUDIT Screen Jamie Quimby.pdfSP Case Pat Stewart.docxSP AUDIT Screen Pat Stewart.pdfEvaluation Tool.docx [file mep_2374-8265.10955-s001.zip › T. Peer Role-Play Case 3-Print GREEN-Provider.docx]

**Green- Role Play Case 3: Joseph (or Josephine)**

**PROVIDER (For the clinician to read):**

**Joseph** is a 35 year old businessman. He presents with epigastric pain and unrelenting nausea which are interfering with his sleep. His symptoms are intermittently relieved by over–the–counter antacids and H2 blockers. His work requires him to travel 3–4 nights per week. He has been particularly busy during the past month. His visit today is prompted by the fact that his symptoms are now interfering with his work. Joseph admits that his stress level has increased recently due to the fact his 5-year marriage seems to be coming to an end.

**OR**

**Josephine** is a 35 year old businesswoman. She presents with epigastric pain and unrelenting nausea which are interfering with her sleep. Her symptoms are intermittently relieved by over–the–counter antacids and H2 blockers. Her work requires her to travel 3–4 nights per week. She has been particularly busy during the past month. Her visit today is prompted by the fact that her symptoms are now interfering with her work. Josephine admits that her stress level has increased recently due to the fact her 5-year marriage seems to be coming to an end.

**ALCOHOL USE QUESTIONS (AUDIT) SCORING:**

Each response from the AUDIT has a score ranging from 0 to 4. The top of each column has a number. That number equals the score value for responses in that column. After a patient has completed the AUDIT, add up each column score, and then sum all five columns for the patient’s score. Below are the scoring guidelines for the AUDIT.

| **Guidelines for Interpretation for AUDIT** | | |
| --- | --- | --- |
| **Score** | **Risk Level** | **Intervention** |
| 0-6 (*Female*)  0-7 (*Male*) | Zone I | Feedback and alcohol education |
| 7-15 (*Female*)  8-15 (*Male*) | Zone II | Brief intervention |
| 16-19 | Zone III | Brief intervention plus brief therapy |
| 20-40 | Zone IV | Brief intervention plus referral to chemical dependency treatment |

**ALCOHOL USE QUESTIONS (AUDIT)**

Drinking alcohol can affect your health and some medications you may take. Please help us provide you with the best medical care by answering the questions below

| **QUESTIONS** | **0** | **1** | **2** | **3** | **4** | **5** | **6** | **Score** |
| --- | --- | --- | --- | --- | --- | --- | --- | --- |
| 1. How often do you have a drink containing alcohol? | Never | Less than monthly | Monthly | Weekly | 2-3 times a week | 4-6 times a week | Daily | **5** |
| 2. How many drinks containing alcohol do you have on a typical day you are drinking? | 1 drink | 2 drinks | 3 drinks | 4 drinks | 5-6 drinks | 7-8  drinks | 10 or more drinks | **4** |
| 3. How often do you have X (5 for men; 4 for women & men over age 65) or more drinks on one occasion? | Never | Less than monthly | Monthly | Weekly | 2-3 times a week | 4-6 times a week | Daily | **6** |
| 4. How often during the last year have you found that you were not able to stop drinking once you had started? | Never | Less than monthly | Monthly | Weekly | Daily or almost daily |  |  | **0** |
| 5. How often during the past year have you failed to do what was expected of you because of drinking? | Never | Less than monthly | Monthly | Weekly | Daily or almost daily |  |  | **2** |
| 6. How often during the past year have you needed a drink first thing in the morning to get yourself going after a heavy drinking session? | Never | Less than monthly | Monthly | Weekly | Daily or almost daily |  |  | **0** |
| 7. How often during the past year have you had a feeling of guilt or remorse after drinking? | Never | Less than monthly | Monthly | Weekly | Daily or almost daily |  |  | **0** |
| 8. How often during the past year have you been unable to remember what happened the night before because you had been drinking? | Never | Less than monthly | Monthly | Weekly | Daily or almost daily |  |  | **0** |
| 9. Have you or someone else been injured because of your drinking? | No |  | Yes, but not in the past year |  | Yes, during the past year |  |  | **0** |
| 10. Has a relative, friend, doctor, or other health care worker been concerned about your drinking and suggested you cut down? | No |  | Yes, but not in the past year |  | Yes, during the past year |  |  | **0** |
|  | | | | | | | **Total *16*** | |
