# Supplementary material for: Standardized Patient Simulation Using SBIRT (Screening, Brief Intervention, and Referral for Treatment) as a Tool for Interprofessional Learning
Source: MedEdPORTAL. 2020 Sep 11;16:10955. doi: 10.15766/mep_2374-8265.10955 (PMC7485913; doi:10.15766/mep_2374-8265.10955)
Supplement: Supplementary file 1 — Educational Objectives.docxAdministrative Instructions Prior to Session.docxStudent Overview of SBIRT Components - Email Prior.docxStudent Prep - ADEPT Video.mp4AUDIT Screening Tool - Email and Print.docxDemonstration - SBIRT Colorado.mp4Faculty Overview and Agenda.docxSBIRT Slides for Live Session.pptxFaculty Script for Slide Presentation.docxSBIRT Pocket Card - Print.pdfStudent Agenda - Print.docxPeer Role-Play Case 1-Print ORANGE-Observer.docxPeer Role-Play Case 1-Print ORANGE-Patient.docxPeer Role-Play Case 1-Print ORANGE-Provider.docxPeer Role-Play Case 2-Print BLUE-Observer.docxPeer Role-Play Case 2-Print BLUE-Patient.docxPeer Role-Play Case 2-Print BLUE-Provider.docxPeer Role-Play Case 3-Print GREEN-Observer.docxPeer Role-Play Case 3-Print GREEN-Patient.docxPeer Role-Play Case 3-Print GREEN-Provider.docxSP Case Jamie Quimby.docxSP AUDIT Screen Jamie Quimby.pdfSP Case Pat Stewart.docxSP AUDIT Screen Pat Stewart.pdfEvaluation Tool.docx [file mep_2374-8265.10955-s001.zip › U. SP Case Jamie Quimby.docx]

SP Case Jamie Quimby

MedEdPORTAL Standardized Patient Case Development Tool

Date: 4/12/2019

Primary Case Author: adapted from materials provided by SAMHSA

Secondary Case Author: Megan Willson

Standardized Patient Educator: Dawn DePriest

Name of Case: Jamie Quimby

Name of educational and or assessment activity: SBIRT session

Patient Name: Jamie Quimby

Chief Complaint: follow-up blood pressure

Most likely Diagnosis and Differential with rationale from history and/or physical exam: Alcohol misuse

Challenge question:

Domains: Check all that apply

- Professionalism
- Communication and Interpersonal skills
- Medical History
- Physical exam
- Shared Decision Making
- Patient Education
- Clinical Reasoning
- Documentation
- Handoff
- Presentation
- Other:

Type and level of learner: interprofessional healthcare students

Case Objectives: please list specific objectives for each of the domains you have checked above:

1. Apply an evidence-based tool (SBIRT) to demonstrate a brief intervention in an interprofessional setting with a standardized patient.

2. Demonstrate giving and receiving timely, instructive feedback between team members regarding their simulated interactions.

| SETTING: outpatient, in patient, ED, home, nursing home, rehab, group etc. | Outpatient clinic |
| --- | --- |
| PATIENT PROFILE: Information about the “patient” that helps select an SP and helps the learner get an understanding of them as a person. SP will know more information about the patient than learner will ever ask but allows SP to portray a fully developed patient personality. If none of the items below are particulars for the case please write “all may be used.” | |
| Age range | 40-50 |
| Religious/spiritual background | all may be used |
| Sex (e.g., male, female, intersex, transwoman, transman) | all may be used |
| Sexual Orientation (e.g., heterosexual, lesbian, gay, bisexual, pansexual, queer, asexual) | all may be used |
| Gender expression (e.g., man, woman, gender queer) | all may be used |
| Race/ethnicity: | all may be used |
| Physical description (e.g., BMI, height range) | all may be used |
| Physical limitations | none |
| Patient appearance (e.g., disheveled, hospital gown, business casual, casual) | Dressed casually, appears to be a middle aged man or woman |
| Moulage + location (e.g., none, bruises, scars, body piercing, tattoos) | none |
| Affect (e.g., pleasant, cooperative) | Pleasant and cooperative |
| Family group (e.g., who is family, who they live with) | Married to your spouse for almost 20 years.  Son in high school. |
| Education | High school or some college |
| Level of health literacy | Adequate |
| Employment, if any - present and past, noting any current stresses | You recently lost your job as an administrative assistant in an office |
| Home/homeless - type of dwelling, number of stories, owned or rented | You rent a two bedroom house in a suburban middle class neighborhood |
| Financial situation- any current stresses | Recently lost your job  You have been arguing with your spouse about financial worries a lot lately |
| Insurance Status (e.g., un/under/insured, public/private, HMO/PPO) | Insured through husband’s job |
| Habits (i.e., diet, exercise, caffeine, smoking, alcohol, drugs) | You drink alcohol (more details below).  You smoked for 20 years, 1 pack a day. You quit two years ago. You are currently not smoking.  You do not currently use recreational drugs. You have used marijuana and cocaine in the past but not for over 20 years |
| Activities (i.e., hobbies, sports, clubs, friends) | You don’t have time for “fun” activities – you are too stressed |
| Typical day - what is the usual daily routine | Up until last week you worked Monday-Friday until 5pm, then came home to your spouse and teenage son. Since losing your job, you’ve been working on projects around the house and filling out job applications online. |

| CASE INFORMATION | |
| --- | --- |
| Chief Concern: What the patient will say when greeted by the student. The patient’s primary reason for seeking medical care often stated in his/own words. | “My blood pressure was high at my last appointment a few weeks ago.” |
| Additional Concerns: Other, if any, concerns the patient has today (i.e., symptoms, requests, expectations, etc.) that will become part of set agenda. | You filled out a questionnaire (the AUDIT) in the waiting room about your alcohol use. This is what your interviewer is going to want to talk about. |
|  | |
| THE PATIENT STORY: The SP will be asked to tell their symptom story and the personal and emotion impact for each of their concerns. You will want to write this is the patient voice. The symptom story should be able to answer this question: “Tell me more about [chief concern/additional concern], starting at the beginning and bringing me up to now.”  The personal context should be able to answer questions concerning the broader personal/psychosocial context of symptoms, especially the patient beliefs/attributions.  The emotional context should be able to ask how are you doing with this, how does this make you feel, how has this affected you emotionally? IMPACT: How has this affected your life? How has this been for your family? | Over the past six months you admit you’ve had a few more glasses of wine a day than usual.  Drinking in the evening helps you cope with “life” and “stress”. You have been arguing with your spouse a lot about financial worries and your son’s behavior. Your son was recently expelled from high school after being found with marijuana on school grounds.  You used to only drink 2 glasses of wine per night but lately have been drinking 3 to 4 glasses.  Last week, you finished a bottle of wine yourself one evening. Your spouse found you asleep in the bathroom floor in the middle of the night.  Your spouse did not know this but you also had a few shots of Sambuca (hard alcohol) that night.  Your spouse is concerned you “passed out” in the bathroom and when you drink you become more “cranky”.  You have not been monitoring your blood pressure at home.  You have been having mild headaches in the morning, which you attribute to “stress”.  You have gained 15 lbs over the past year.  AUDIT ANSWERS: see questionnaire, but if asked about any answers:   1. *How often you have a drink containing alcohol?* **Hardly ever skip a day** 2. *How many drinks containing alcohol do you have on a typical day when you are drinking?* **Usually 3 or 4** 3. *How often do you have six or more drinks on one occasion?* **Maybe once a week** 4. *How often during the last year have you found that you were not able to stop drinking once you had started?* **Never, I know I could stop** 5. *How often during the last year have you failed to do what was normally expected of you because of drinking?* **Never** 6. *How often during the last have you needed a first drink in the morning to get yourself going after a heavy drinking session?* **I never drink in the morning** 7. *How often during the last year have you had a feeling of guilt or remorse after drinking?* **A couple times I yelled at my son. I don’t think I would have said those things if I wasn’t drinking.** 8. *How often during the last year have you been unable to remember what happened the night before because of your drinking?* **I don’t know how I ended up in the bathroom last week.** 9. *Have you or someone been injured because of your drinking?* **No** 10. *Has a relative, friend, doctor or other healthcare worked been concerned about your drinking or suggested you cut down?* **Spouse concerned I fell asleep in bathroom, and thinks drinking makes me “cranky”** |
|  | |
| HISTORY OF PRESENT ILLNESS: Although some of the HPI will be given in the patient’s symptom story, the learners will expand the story during the direct question section. Below describe the detailed history, usually about the chief concern, which the student must develop in order to make a useful assessment of the problem: | |
|  | |
| Onset (when; gradual or sudden) | NA |
| Setting (what was going on or where was patient when symptoms first noticed?) | NA |
| Duration (how long) | NA |
| Time relationships (frequency, constant or intermittent) | NA |
| Location | NA |
| Radiation | NA |
| Quality | NA |
| Amount | NA |
| Aggravated by what | NA |
| Relieved by what | NA |
| Associated with what | NA |
| Attitude (what does the patient think is the problem, and how does he/she feel about it) | *When the student wants to discuss making a change, you will have a different response for each student in the triad.*  Round 1: Amicable to treatment (you agree you want to make a change)   - Permission to discuss alcohol/drug use: **Yes**, you are open to listen - Readiness to change: You answer **5 or higher out of 10** on the readiness /confidence ruler. You have reasons to change (choose something the students suggest, such as your son, your spouse, and/or your blood pressure) - You are **receptive to cutting back** on your drinking if suggestions are provided. If they suggest it, you are open to cutting back to only drinking on weekends, or having fewer drinks per night - You **are willing** to schedule a follow-up appointment with the team if requested to further discuss your drinking.   Round 2: Minimizing/In Denial (You do not see that there is an issue)   - Permission to discuss alcohol/drug use: **Yes, BUT** there is nothing to really talk about. You don’t think there is any connection between your increase in alcohol use and problems at home (fighting with your spouse or your son getting caught with drugs) or losing your job or your high blood pressure. Changing your drinking won’t change those, and drinking helps you cope. - Readiness to change: You answer **lower than 5 out of 10** on the readiness /confidence ruler. There is no reason to change if there is no problem. “They need to change (your spouse, son, or employer), not me.” - You are **not receptive** to cutting back on your drinking if suggestions are provided - If suggested: you might be willing to schedule a follow-up appointment with the team someday IF you feel like you need it.   Round 3: Resistant to Change (too busy, too many other things going on)   - Permission to discuss alcohol/drug use: **Yes, BUT** you don’t really have time – you just need your blood pressure taken care of. - Readiness to change: You answer **zero out of 10** on the readiness /confidence ruler. You have to figure things out with your spouse, your son, and get a new job. That’s too much stress to throw something new at you. - You are **not receptive** to cutting back on your drinking and you don’t have time with everything else going on. Right now having a glass of wine at night is the *only* thing that is helping you deal with your problems/stress. - You are **not willing** to schedule a follow-up appointment with the team if requested to further discuss your drinking; you can’t possible fit one more thing into your schedule. |
| Overall course | NA |
| REVIEW OF SYSTEMS: Significant positives and negatives | |
|  |  |
|  |  |
|  |  |
|  |  |
| General Rule of Thumb: If the student asks a question that has not been addressed in these training materials, your answer should be “no,” (meaning that you have not had that symptom or problem). The student might ask your personal or social history questions that have not been addressed in these materials. In this case, you answer appropriately for the character you are portraying  Dealing with Multiple Questions:   - If the student asks a multiple or compound question consisting of 2 questions (such as “Have you had chest pain or shortness of breath?”), you can answer BOTH questions, but must provide a clear answer for each component separately even if the answer for each is the same. In other words, do not just answer “No” to a compound question, say: “No, I haven’t had chest pain or shortness of breath”. - If the student rattles off a list of 3 or more questions, answer only the last question asked (make it clear that you are answering only that one question). For example, the student may ask “do you drink alcohol, smoke, or use street drugs?”, in which case you would answer “No, I don’t use street drugs.” | |
| Past medical history | High blood pressure |
| Medication allergies (Name and reaction) | Sulfa - hives |
| Environmental allergies (Name and reaction) | none |
| Illnesses | none |
| Vaccinations | “I’m up to date as far as I know” |
| Surgeries | none |
| Accidents/ injuries/ trauma | none |
| Hospitalization | none |
|  | |
| Inclusive sexual and reproductive history | |
| Sexual practices  Sexual partners  Protection: Use of safer sex practices  Use of birth control if appropriate  Risk of intimate partner violence | You are sexually active with your spouse, usually once or twice a week. You don’t have any other partners nor does your spouse as far as you know. You feel safe at home, and your spouse has never hurt you. |
| Ob/GYN HISTORY  (*if SP is female*) | Age of onset of menses: 11  Age of menopause: NA  Number of pregnancies: 1  Number of live births: 1  Number of miscarriages: 0  Number of abortions: 0 |
| Medications | Prescription/dose/reason  Lisinopril 20mg orally every morning, for high blood pressure  Over the counter/dose/reason  You take occasional over the counter advil for headaches |
| Immunizations (if asked) | - Tetanus – “I’m up to date as far as I know” - Flu – “I got one this year” - Hepatitis – “I don’t know” - Pneumovax – “I don’t know” - HPV – “No, I haven’t had that one” - Other |
| Tobacco products:   - Cigarettes – yes, in past - Cigar – no - Pipe – no - Chew – no - E-cigarettes – no | Cigarettes in the past – You started in 1996, quit 2016. You smoked 1 pack per day. You quit when you met your spouse. You do not smoke now. |
| Alcohol   - Beer - Wine - Liquor - Other | Yes - See above for details |
| Drugs   - Weed - Cocaine - Heroin - Meth - Other - IV - Inhalants - Other | Never |
| Diet (describe) | “I try to eat a balanced diet, but produce is expensive” |
| Exercise (describe) | “I don’t have time to exercise” |
| List any other important social history or information important to this case |  |
| Family history |  |
| Mother, Father, Siblings, Grandparents, and other significant findings. | You do not know about any family history of high blood pressure. Father drank alcohol but was never a problem |
|  |  |
| Physical Exam- List exam maneuvers expected for this case and any abnormal findings that SP will simulate. (tenderness, hyper-hypo reflex, rebound, weakness etc. )  No physical exam | |
|  | |
