# Supplementary material for: Standardized Patient Simulation Using SBIRT (Screening, Brief Intervention, and Referral for Treatment) as a Tool for Interprofessional Learning
Source: MedEdPORTAL. 2020 Sep 11;16:10955. doi: 10.15766/mep_2374-8265.10955 (PMC7485913; doi:10.15766/mep_2374-8265.10955)
Supplement: Supplementary file 1 — Educational Objectives.docxAdministrative Instructions Prior to Session.docxStudent Overview of SBIRT Components - Email Prior.docxStudent Prep - ADEPT Video.mp4AUDIT Screening Tool - Email and Print.docxDemonstration - SBIRT Colorado.mp4Faculty Overview and Agenda.docxSBIRT Slides for Live Session.pptxFaculty Script for Slide Presentation.docxSBIRT Pocket Card - Print.pdfStudent Agenda - Print.docxPeer Role-Play Case 1-Print ORANGE-Observer.docxPeer Role-Play Case 1-Print ORANGE-Patient.docxPeer Role-Play Case 1-Print ORANGE-Provider.docxPeer Role-Play Case 2-Print BLUE-Observer.docxPeer Role-Play Case 2-Print BLUE-Patient.docxPeer Role-Play Case 2-Print BLUE-Provider.docxPeer Role-Play Case 3-Print GREEN-Observer.docxPeer Role-Play Case 3-Print GREEN-Patient.docxPeer Role-Play Case 3-Print GREEN-Provider.docxSP Case Jamie Quimby.docxSP AUDIT Screen Jamie Quimby.pdfSP Case Pat Stewart.docxSP AUDIT Screen Pat Stewart.pdfEvaluation Tool.docx [file mep_2374-8265.10955-s001.zip › V. SP AUDIT Screen Jamie Quimby.pdf]

Jamie Quimby

The Alcohol Use Disorders Identification Test: Self-Report Version

PATIENT: Because alcohol use can affect your health and can interfere with certain medications and treatments, it is important that we ask some questions about your use of alcohol. Your answers will remain confidential so please be honest. Place an X in one box that best describes your answer to each question.

Questions	0	1	2	3	4	
1. How often do you have a drink containing alcohol?	Never	Monthly or less	2-4 times a month	2-3 times a week	4 or more times a week	
2. How many drinks containing alcohol do you have on a typical day when you are drinking?	1 or 2	3 or 4	5 or 6	7 to 9	10 or more	
3. How often do you have six or more drinks on one occasion?	Never	Less than monthly	Monthly	Weekly	Daily or almost daily	
4. How often during the last year have you found that you were not able to stop drinking once you had started?	Never	Less than monthly	Monthly	Weekly	Daily or almost daily	
5. How often during the last year have you failed to do what was normally expected of you because of drinking?	Never	Less than monthly	Monthly	Weekly	Daily or almost daily	
6. How often during the last year have you needed a first drink in the morning to get yourself going after a heavy drinking session?	Never	Less than monthly	Monthly	Weekly	Daily or almost daily	
7. How often during the last year have you had a feeling of guilt or remorse after drinking?	Never	Less than monthly	Monthly	Weekly	Daily or almost daily	
8. How often during the last year have you been unable to remember what happened the night before because of your drinking?	Never	Less than monthly	Monthly	Weekly	Daily or almost daily	
9. Have you or someone else been injured because of your drinking?	No		Yes, but not in the last year		Yes, during the last year	
10. Has a relative, friend, doctor, or other health care worker been concerned about your drinking or suggested you cut down?	No		Yes, but not in the last year		Yes, during the last year	
					**Total**	

Adapted from: Babor T, et al. World Health Organization. 2001. AUDIT: the Alcohol Use Disorders Identification Test: guidelines for use in primary health care. 2nd ed. World Health Organization.
