# Supplementary material for: Standardized Patient Simulation Using SBIRT (Screening, Brief Intervention, and Referral for Treatment) as a Tool for Interprofessional Learning
Source: MedEdPORTAL. 2020 Sep 11;16:10955. doi: 10.15766/mep_2374-8265.10955 (PMC7485913; doi:10.15766/mep_2374-8265.10955)
Supplement: Supplementary file 1 — Educational Objectives.docxAdministrative Instructions Prior to Session.docxStudent Overview of SBIRT Components - Email Prior.docxStudent Prep - ADEPT Video.mp4AUDIT Screening Tool - Email and Print.docxDemonstration - SBIRT Colorado.mp4Faculty Overview and Agenda.docxSBIRT Slides for Live Session.pptxFaculty Script for Slide Presentation.docxSBIRT Pocket Card - Print.pdfStudent Agenda - Print.docxPeer Role-Play Case 1-Print ORANGE-Observer.docxPeer Role-Play Case 1-Print ORANGE-Patient.docxPeer Role-Play Case 1-Print ORANGE-Provider.docxPeer Role-Play Case 2-Print BLUE-Observer.docxPeer Role-Play Case 2-Print BLUE-Patient.docxPeer Role-Play Case 2-Print BLUE-Provider.docxPeer Role-Play Case 3-Print GREEN-Observer.docxPeer Role-Play Case 3-Print GREEN-Patient.docxPeer Role-Play Case 3-Print GREEN-Provider.docxSP Case Jamie Quimby.docxSP AUDIT Screen Jamie Quimby.pdfSP Case Pat Stewart.docxSP AUDIT Screen Pat Stewart.pdfEvaluation Tool.docx [file mep_2374-8265.10955-s001.zip › W. SP Case Pat Stewart.docx]

SP case Pat Stewart

MedEdPORTAL Standardized Patient Case Development Tool

Date: 4/12/2019

Primary Case Author: Megan Willson

Secondary Case Author: Janelle Clauser

Standardized Patient Educator: Dawn DePriest

Name of Case: Pat Stewart

Name of educational and or assessment activity: SBIRT session

Patient Name: Pat Stewart

Chief Complaint: “I drank a lot and the police brought me to the hospital because I had a cut on my arm”

Most likely Diagnosis and Differential with rationale from history and/or physical exam: alcohol misuse

Challenge question:

Domains: Check all that apply

- Professionalism
- Communication and Interpersonal skills
- Medical History
- Physical exam
- Shared Decision Making
- Patient Education
- Clinical Reasoning
- Documentation
- Handoff
- Presentation
- Other:

Type and level of learner:

Case Objectives: please list specific objectives for each of the domains you have checked above:

1. Apply an evidence-based tool (SBIRT) to demonstrate a brief intervention in an interprofessional setting with a standardized patient.

2. Demonstrate giving and receiving timely, instructive feedback between team members regarding their simulated interactions

| SETTING: outpatient, in patient, ED, home, nursing home, rehab, group etc. | Inpatient hospital, awaiting discharge. You have been admitted for the last 8 days. |
| --- | --- |
| PATIENT PROFILE: Information about the “patient” that helps select an SP and helps the learner get an understanding of them as a person. SP will know more information about the patient than learner will ever ask but allows SP to portray a fully developed patient personality. If none of the items below are particulars for the case please write “all may be used.” | |
| Age range | age range 50-60 |
| Religious/spiritual background | All may be used |
| Sex (e.g., male, female, intersex, transwoman, transman) | All may be used |
| Sexual Orientation (e.g., heterosexual, lesbian, gay, bisexual, pansexual, queer, asexual) | All may be used |
| Gender expression (e.g., man, woman, gender queer) | All may be used |
| Race/ethnicity: | All may be used |
| Physical description (e.g., BMI, height range) | All may be used |
| Physical limitations | none |
| Patient appearance (e.g., disheveled, hospital gown, business casual, casual) | Dressed in comfortable clothing |
| Moulage + location (e.g., none, bruises, scars, body piercing, tattoos) | Bandage on arm where the laceration was sutured |
| Affect (e.g., pleasant, cooperative) | Responsive to questions – conversational  You are under a lot of stress because you just lost your home and job  You do not feel like you are going to harm yourself or someone else |
| Family group (e.g., who is family, who they live with) | Not married – your spouse divorced you while you were in prison, 3 years ago |
| Education | Completed high school |
| Level of health literacy | Poor |
| Employment, if any - present and past, noting any current stresses | You were working under the table for a construction company but they fired you a month ago. They said it was because of drinking which makes you angry because you never showed up to work drunk. |
| Home/homeless - type of dwelling, number of stories, owned or rented | You had an apartment you were barely making the rent on. After you lost your job you couldn’t pay rent so you’ve been staying with different buddies, mostly sleeping on couches. |
| Financial situation- any current stresses | You recently lost your job. You do not have a car. |
| Insurance Status (e.g., un/under/insured, public/private, HMO/PPO) | You do not have insurance. |
| Habits (i.e., diet, exercise, caffeine, smoking, alcohol, drugs) |  |
| Activities (i.e., hobbies, sports, clubs, friends) | You have a few buddies you drink with |
| Typical day - what is the usual daily routine | You are vague – “this or that.” |

| CASE INFORMATION | |
| --- | --- |
| Chief Concern: What the patient will say when greeted by the student. The patient’s primary reason for seeking medical care often stated in his/own words. | “I drank a lot and the police brought me to the hospital because I had a cut on my arm” |
| Additional Concerns: Other, if any, concerns the patient has today (i.e., symptoms, requests, expectations, etc.) that will become part of set agenda. | - You just lost your home recently and have no car. - You quit taking your medications a few months ago because they made me feel “off”. |
|  | |
| THE PATIENT STORY: The SP will be asked to tell their symptom story and the personal and emotion impact for each of their concerns. You will want to write this is the patient voice. The symptom story should be able to answer this question: “Tell me more about [chief concern/additional concern], starting at the beginning and bringing me up to now.”  The personal context should be able to answer questions concerning the broader personal/psychosocial context of symptoms, especially the patient beliefs/attributions.  The emotional context should be able to ask how are you doing with this, how does this make you feel, how has this affected you emotionally? IMPACT: How has this affected your life? How has this been for your family? | You are waiting to go home after being in the hospital for 8 days for “DT’s”.  The doctors told you that someone found you at a bus stop with a fifth of whiskey and a large gash on your arm. They brought you into the ER because of your arm, but you don’t know how you got it and don’t remember them stitching you up. They told you that you started having DT’s or “shakes” (detox tremors from alcohol withdrawl) in the ER so they admitted you and you’ve been here since. You only remember the last couple of days – you have no recollection of the first 6 days in the hospital. Now that that’s over you think you’re good.  You’ve been drinking more and more since you got released from prison 6 months ago.  You were sober for 6 years in prison, but drank heavily before that.  Drinking helps you cope with “life” and “stress”.  You used to drink 12-18 beers a day before, but now you’ve switched to drinking hard liquor.  AUDIT ANSWERS: see questionnaire, but if asked about any answers:   1. How often you have a drink containing alcohol? Hardly ever skip a day 2. How many drinks containing alcohol do you have on a typical day when you are drinking? A fifth or more depending on how much I can get 3. How often do you have six or more drinks on one occasion? Maybe once a week 4. How often during the last year have you found that you were not able to stop drinking once you had started? Probably once a month 5. How often during the last year have you failed to do what was normally expected of you because of drinking? Never – no one who expects anything 6. How often during the last have you needed a first drink in the morning to get yourself going after a heavy drinking session? Most days 7. How often during the last year have you had a feeling of guilt or remorse after drinking? Sometimes I wish I didn’t drink so much 8. How often during the last year have you been unable to remember what happened the night before because of your drinking? Many this last few months. 9. Have you or someone been injured because of your drinking? No 10. Has a relative, friend, doctor or other healthcare worked been concerned about your drinking or suggested you cut down? All the providers here at the hospital. I don’t have really any close friends. Just people that I drink with. |
| HISTORY OF PRESENT ILLNESS: Although some of the HPI will be given in the patient’s symptom story, the learners will expand the story during the direct question section. Below describe the detailed history, usually about the chief concern, which the student must develop in order to make a useful assessment of the problem: | |
|  | |
| Onset (when; gradual or sudden) | - You started drinking when you were in junior high |
| Setting (what was going on or where was patient when symptoms first noticed?) | - You had DT’s in the ER when you arrived 8 days ago - You’ve been drinking more and more since you got released from prison 6 months ago |
| Duration (how long) | NA |
| Time relationships (frequency, constant or intermittent) | - Heavy drinking history prior to going to prison 6 years ago - No drinking in prison for 6 years - Increased drinking for last 6 months |
| Location | NA |
| Radiation | NA |
| Quality | NA |
| Amount | - Depends on what you can get. Usually a fifth of hard liquor or a few “tall boy” (16-24 oz) beers |
| Aggravated by what | Drinking helps you **cope with “life” and “stress”**  Your friends all drink |
| Relieved by what |  |
| Associated with what |  |
| Attitude (what does the patient think is the problem, and how does he/she feel about it) | *When the student wants to discuss making a change, you will have a different response for each student in the triad.*  Round 1: Amicable to treatment (you agree you want to make a change)   - Receptive to talking with the team about your drinking - On the ***readiness scale your motivation*** is “7 out of 10” to change. - Unsure what is the best way. Everyone on streets and all my friends drink even more than me, but I don’t want this to happen again. The withdrawal almost killed me.   Round 2: Minimizing/In Denial (you do not see that there is an issue)   - On the ***readiness scale your motivation*** is “3 out of 10” to change. - I don’t see why I need to cut down. I just got admitted to the hospital because I had that cut. If they had just given me beer while I was in the hospital like the time before I would have been fine and not had shakes. - If I cut down, I won’t need to quit alcohol. I’ll just stick with beer, that way I won’t go through withdrawal - If suggested: receptive to coming in for a follow-up appointment with the team in a few weeks. Not interested in behavioral counseling.   Round 3: Resistant to change (too busy, many other things going on)   - On the ***readiness scale your motivation*** is “3 out of 10” to change. - Nothing will ever work. I have done it all before and I still ended up here - If suggested: make up excuses why it won’t work – ie “I already tried that” or “that never works for nobody” - I don’t have anybody to change for. This is what I enjoy doing. |
| Overall course |  |
| REVIEW OF SYSTEMS: Significant positives and negatives | |
|  |  |
|  |  |
|  |  |
|  |  |
| **General Rule of thumb:**   - If the student asks a question that has not been addressed in these training materials, your answer should be “no,” (meaning that you have not had that symptom or problem). The student might ask your personal or social history questions that have not been addressed in these materials. In this case, you answer appropriately for the character you are portraying.   **Dealing with Multiple Questions:**   - If the student asks a multiple or compound question consisting of 2 questions (such as “Have you had chest pain or shortness of breath?”), you can answer BOTH questions, but must provide a clear answer for each component separately even if the answer for each is the same. In other words, do not just answer “No” to a compound question, say: “No, I haven’t had chest pain or shortness of breath”. - If the student rattles off a list of 3 or more questions, answer only the last question asked (make it clear that you are answering only that one question). For example, the student may ask “do you drink alcohol, smoke, or use street drugs?”, in which case you would answer “No, I don’t use street drugs | |
| Past medical history |  |
| Medication allergies (Name and reaction) | Sulfa (rash) |
| Environmental allergies (Name and reaction) | none |
| Illnesses | Bipolar disorder |
| Vaccinations | “I’m up to date as far as I know” |
| Surgeries | none |
| Accidents/ injuries/ trauma | The healing laceration on your arm now |
| Hospitalization | Admitted to psychiatry once 8 years ago, started on psych meds but don’t recall what they were. You aren’t taking any now. |
|  | |
| Inclusive sexual and reproductive history | |
| Sexual practices  Sexual partners  Protection: Use of safer sex practices  Use of birth control if appropriate  Risk of intimate partner violence | Any can be used |
| Ob/GYN HISTORY  *(IF SP is female)* | Age of onset of menses: 12  Age of menopause: early 40s  Number of pregnancies: none  Number of live births: none  Number of miscarriages: none  Number of abortions: none |
| Medications | You quit taking your medications a few months ago because they made me feel “off”.  You take an occasional over-the counter Tylenol of Motrin for aches/pains |
| Immunizations | “I’m up to date as far as I know” |
| Tobacco products:  Cigarettes - yes | You smoked for 20 years, 1 pack per day. You quit 2 years ago and haven’t smoked since. |
| Alcohol  Beer - yes  Liquor - yes | Current everyday drinking (see above) |
| Drugs  Meth – yes  Other drugs – no | You have used meth in the past but not on regular basis. You last used about 3 months ago.  You don’t use any other drugs. |
| Diet (describe) | You eat a lot of fast food. |
| Exercise (describe) | You don’t exercise. |
| List any other important social history or information important to this case |  |
| Family history |  |
| Mother, Father, Siblings, Grandparents, and other significant findings. | none |
|  |  |
| Physical Exam- List exam maneuvers expected for this case and any abnormal findings that SP will simulate. (tenderness, hyper-hypo reflex, rebound, weakness etc. )  No physical exam | |
| PHYSICAL EXAM FINDINGS |  |
| 1. Written in layman’s terms |  |
| 1. General appearance- affect, appearance, position of patient at opening (i.e. sitting, laying down, holding abdomen etc.) |  |
| 1. Vital signs |  |
| 1. Specific findings and affect |  |
| 1. Response to certain physical movements |  |
|  |  |
| DIAGNOSIS AND DIFFERENTIAL |  |
| Diagnosis with support from positive and negative history and PE findings |  |
| Differential with support from positive and negative history and PE findings |  |
|  |  |
| MANAGEMENT OR DIAGNOSITIC PLAN |  |
|  |  |
| PROFESSIONALISM ISSUES OR CHALLENGES: |  |
