# Supplementary material for: Standardized Patient Simulation Using SBIRT (Screening, Brief Intervention, and Referral for Treatment) as a Tool for Interprofessional Learning
Source: MedEdPORTAL. 2020 Sep 11;16:10955. doi: 10.15766/mep_2374-8265.10955 (PMC7485913; doi:10.15766/mep_2374-8265.10955)
Supplement: Supplementary file 1 — Educational Objectives.docxAdministrative Instructions Prior to Session.docxStudent Overview of SBIRT Components - Email Prior.docxStudent Prep - ADEPT Video.mp4AUDIT Screening Tool - Email and Print.docxDemonstration - SBIRT Colorado.mp4Faculty Overview and Agenda.docxSBIRT Slides for Live Session.pptxFaculty Script for Slide Presentation.docxSBIRT Pocket Card - Print.pdfStudent Agenda - Print.docxPeer Role-Play Case 1-Print ORANGE-Observer.docxPeer Role-Play Case 1-Print ORANGE-Patient.docxPeer Role-Play Case 1-Print ORANGE-Provider.docxPeer Role-Play Case 2-Print BLUE-Observer.docxPeer Role-Play Case 2-Print BLUE-Patient.docxPeer Role-Play Case 2-Print BLUE-Provider.docxPeer Role-Play Case 3-Print GREEN-Observer.docxPeer Role-Play Case 3-Print GREEN-Patient.docxPeer Role-Play Case 3-Print GREEN-Provider.docxSP Case Jamie Quimby.docxSP AUDIT Screen Jamie Quimby.pdfSP Case Pat Stewart.docxSP AUDIT Screen Pat Stewart.pdfEvaluation Tool.docx [file mep_2374-8265.10955-s001.zip › Y. Evaluation Tool.docx]

**Interprofessional Activity Evaluation Tool**

**Please rate the extent of your agreement with each of the following statements:**

| Value/Importance of Working with Interdisciplinary Team | Strongly  Disagree | Disagree | Neutral | Agree | Strongly  Agree |  | Question reference |
| --- | --- | --- | --- | --- | --- | --- | --- |
| SBIRT is an effective activity for interprofessional learning. |  |  |  |  |  |  | IPEC CC |
| SBIRT is an effective activity for developing mutual respect and shared value amongst a team of professionals from different disciplines. |  |  |  |  |  |  | IPEC CC |
| SBIRT is an effective activity for developing skills to communicate with other health professionals, patients, families, or communities in a responsive and responsible manner. |  |  |  |  |  |  | IPEC CC |
| Working with another discipline of students enhances my education. |  |  |  |  |  |  | SPICE 1 |
| My role within the interdisciplinary team is clearly defined. |  |  |  |  |  |  | SPICE 2 |
| Health outcomes are improved when patients are treated by a team of professionals from different disciplines. |  |  |  |  |  |  | SPICE 3 |
| Patient satisfaction is improved when patients are treated by a team of professionals from different disciplines. |  |  |  |  |  |  | SPICE 4 |
| Participating in educational experiences with another discipline of students enhances my future ability to work on an interdisciplinary team. |  |  |  |  |  |  | SPICE 5 |
| All health professions students should be educated to establish collaborative relationships with members from other disciplines. |  |  |  |  |  |  | SPICE 6 |
| I understand the roles of other professionals within the interdisciplinary team. |  |  |  |  |  |  | SPICE 7 |
| Clinical simulations are the ideal place within their respective curricula for health professional students to interact. |  |  |  |  |  |  | SPICE 8 |
| Health Professionals should collaborate in teams |  |  |  |  |  |  | SPICE 9 |
| During their education, students from different healthcare disciplines should be involved in teamwork in order to understand their respective roles. |  |  |  |  |  |  | SPICE 10 |

2. How ***useful*** was this activity to your interprofessional development?

1=Not at all useful, 2=slightly useful, 3=somewhat useful, 4=very useful, 5=extremely useful

3. Based on your experience, what do you believe are the ***benefits*** of an ***interprofessional team approach*** to caring for patients with substance abuse behaviors? Open Comment box:

*IPEC CC = Interprofessional Education Collaborative Core Competency*

*SPICE = Student Perceptions of Interprofessional Clinical Education tool*

**Interprofessional Activity Evaluation Tool Documentation**

The first three questions evaluate core competencies (CoreC): Overall (CoreC-Overall); Values and Ethics (CoreC-VE) and Communication and Collaboration (CoreC-CC).

The Students Perceptions of Practitioner Interprofessional Clinical Education (SPICE) tool is a validated and reliable tool, previously published and used by other groups. Approval to use the SPICE tool is given at <https://nexusipe.org/resource-exchange/tss-team-skills-scale>. The modified tool includes all 10 questions across three subscales:

- - 1. Patient Outcomes from Collaborative Practice (SPICE-O)
    2. Interprofessional Teamwork and Team-based Practice (SPICE-T)
    3. Roles/Responsibilities for Collaborative Practice (SPICE-R)

Minor modifications involving word replacement to make the tool applicable to all health professional students were made:

SPICE8-T4:

Original: Clinical rotations are the ideal place within their respective curricula for medical and pharmacy students to interact. Revised: Clinical simulations are the ideal place within their respective curricula for health professional students to interact.

SPICE9-T5:

Original: Physicians and pharmacists should collaborate in teams

Revised: Health Professionals should collaborate in teams

For program evaluation, students are asked about the usefulness of the activity.

The free response question addresses benefits of interprofessional education through the activity.
