# Supplementary material for: Standardized Patient Simulation Using SBIRT (Screening, Brief Intervention, and Referral for Treatment) as a Tool for Interprofessional Learning
Source: MedEdPORTAL. 2020 Sep 11;16:10955. doi: 10.15766/mep_2374-8265.10955 (PMC7485913; doi:10.15766/mep_2374-8265.10955)
Supplement: Supplementary file 1 — Educational Objectives.docxAdministrative Instructions Prior to Session.docxStudent Overview of SBIRT Components - Email Prior.docxStudent Prep - ADEPT Video.mp4AUDIT Screening Tool - Email and Print.docxDemonstration - SBIRT Colorado.mp4Faculty Overview and Agenda.docxSBIRT Slides for Live Session.pptxFaculty Script for Slide Presentation.docxSBIRT Pocket Card - Print.pdfStudent Agenda - Print.docxPeer Role-Play Case 1-Print ORANGE-Observer.docxPeer Role-Play Case 1-Print ORANGE-Patient.docxPeer Role-Play Case 1-Print ORANGE-Provider.docxPeer Role-Play Case 2-Print BLUE-Observer.docxPeer Role-Play Case 2-Print BLUE-Patient.docxPeer Role-Play Case 2-Print BLUE-Provider.docxPeer Role-Play Case 3-Print GREEN-Observer.docxPeer Role-Play Case 3-Print GREEN-Patient.docxPeer Role-Play Case 3-Print GREEN-Provider.docxSP Case Jamie Quimby.docxSP AUDIT Screen Jamie Quimby.pdfSP Case Pat Stewart.docxSP AUDIT Screen Pat Stewart.pdfEvaluation Tool.docx [file mep_2374-8265.10955-s001.zip › _Educational_Summary_Report_10955.pdf]

MedEdPORTAL® The AAMC Journal of Teaching and Learning Resources

Original Publication Open Access

Standardized Patient Simulation Using SBIRT (Screening, Brief Intervention, and Referral for Treatment) as a Tool for Interprofessional Learning

Janelle Clauser, MD*, Barbara B. Richardson, PhD, RN, Tamara Odom-Maryon, PhD, Donna Mann, OTD, MEd, Megan N. Willson, PharmD, Patricia L. Hahn, MN, ARNP, Janet Purath, PhD, ARNP, Erica Tuell, Catrina R. Schwartz, PharmD, Dawn DePriest, DNP, ARNP

*Corresponding author: jclauser@uw.edu

Abstract

Introduction: Substance misuse is a critical social and health care issue, and learning how to effectively screen for misuse and perform a brief intervention is useful for all health care professions. As an intercollegiate, interprofessional group, we developed a mechanism for delivering interprofessional education (IPE) using SBIRT (screening, brief intervention, and referral for treatment) as a tool to identify potential substance misuse. **Methods:** A total of 1,255 students from nursing, pharmacy, medicine, physician assistant, social work, dietetics, and occupational therapy programs participated in the training and evaluation of this IPE experience over 2 academic years. The training incorporated asynchronous SBIRT training, in-person student role-plays, and a standardized patient (SP) interaction. **Results:** A significant majority of participants indicated that this IPE experience enhanced their interprofessional skills (91%), was useful for interprofessional development (79%), was relevant to their career (92%), and would benefit their clients (93%). Faculty debrief sessions supported the efficacy of SBIRT as a platform for IPE. **Discussion:** Students believed that utilizing SBIRT as an interprofessional learning experience enhanced their overall educational experience and assisted with developing interprofessional relationships and that team-based care would lead to improved patient outcomes. Faculty found this learning activity to be effective in developing student insight regarding future professional peers and patient interview skill development through role-plays with peers and SPs.

Keywords
SBIRT, Substance Use, Interprofessional, Multidisciplinary, Simulation, Standardized Patients, Case-Based Learning, Clinical Teaching/Bedside Teaching, Flipped Classroom, Problem-Based Learning, Editor's Choice

Educational Objectives

By the end of this activity, learners will be able to:

1. Describe screening, brief intervention, and referral for treatment (SBIRT) as a valuable tool for multiple health professions for identifying risk of substance misuse and providing a brief, point-of-care intervention.
2. Identify their role in the SBIRT process and compare roles with those of other health professional students (nursing, pharmacy, medicine, social work, occupational therapy, physician assistant, and dietetics).
3. Apply an evidence-based tool (SBIRT) to demonstrate a brief intervention in an interprofessional setting with a standardized patient.
4. Demonstrate giving and receiving timely, instructive feedback between team members regarding their interactions with a simulated patient.

Citation:
Clauser J, Richardson BB, Odom-Maryon T, et al. Standardized patient simulation using SBIRT (screening, brief intervention, and referral for treatment) as a tool for interprofessional learning. *MedEdPORTAL*. 2020;16:10955.
https://doi.org/10.15766/mep_2374-8265.10955

Introduction

Currently, substance misuse is a widespread social and health care issue with implications for all health care professions. The screening, brief intervention, and referral to treatment (SBIRT) technique is an evidence-based tool to identify potential misuse and prevent abuse of alcohol and/or illicit drugs. SBIRT was selected for this interprofessional activity because it incorporates common curricular content, including team-based patient-centered care, motivational interviewing skills, and population health and prevention.

Interprofessional education (IPE) programs require strong faculty commitment and a dedication to ongoing collaboration.[1] The

Copyright © 2020 Clauser et al. This is an open-access publication distributed under the terms of the Creative Commons Attribution license.

goal of IPE is to prepare workforce-ready health care team members who practice collaboratively in order to improve health outcomes.[2] In response to the growing complexity of care, as well as accreditation requirements, health professions programs now integrate teamwork and communication content in their curricula. In order to create meaningful IPE learning opportunities, educators strive to design, implement, and evaluate learning opportunities that simulate patient care scenarios students will likely encounter in clinical settings.

The resource described here drew from lessons learned through a previous interprofessional Health Service and Research Administration–funded program that included faculty development, implementation of an interprofessional team-based program to teach collaborative practice skills using standardized patients (SPs), and multiple assessment and evaluation strategies to provide formative feedback to students.[3] Using SBIRT as the vector for IPE, the goal of this program was to provide a meaningful interprofessional learning activity for students ($N = 1,255$) in medicine, nursing (BSN and DNP), pharmacy, physician assistant, social work, dietetics, and occupational therapy programs.

Other *MedEdPORTAL* publications have shared SBIRT cases for medical resident training using SPs[4] or using SBIRT to facilitate interprofessional instruction of postgraduate health care professionals.[5,6] In this module, we offer a guide for presession independent learning followed by SBIRT cases for a single in-person session of peer role-play practice and SP interactions, with students from seven different health professions as the target learners.

Methods

Design

This project was reviewed by the Washington State University Institutional Review Board (the host university), which determined the project satisfied the criteria for exempt research.

We designed this SBIRT educational module for teaching interprofessional student learners from concept to skills application. We assembled an interprofessional team comprising medicine, nursing (BSN and DNP), pharmacy, physician assistant, social work, dietetics, and occupational therapy faculty to design, implement, and evaluate the educational module and learning objectives (Appendix A). Student participants represented the same seven professions. These programs were included because they were professions likely to use motivational interviewing skills, specifically SBIRT, in collaborative care practice settings. We chose substance misuse as the focus of the program because of the ubiquitous impact of alcohol and illicit drugs in all health care settings and all communities. After evaluating numerous options, we selected SBIRT because it was evidence based and applied strategies of preventive care, motivational interviewing, and team-based patient-centered care. All faculty who developed and delivered content completed a 4-hour SBIRT training provided by a certified SBIRT educator.

For student participants, the educational module consisted of a required 2-hour online informational component (completed at any point prior to the live session) and a 2-hour in-person application session. Participation was required as a class assignment in each profession-specific program.

Preclass component: A subset of faculty reviewed available online programs for quality and applicability across multiple disciplines, as well as ease of registration and use. Trainings from Medscape (which later became unavailable) and SBIRT Oregon were selected.[7] These two trainings were similar in defining SBIRT, introducing screening tools for identifying drug and alcohol misuse, demonstrating a brief intervention, and discussing impacts and benefits of using SBIRT. Prior to the in-person session, all students were required to complete the online preclass component and watch a video[8] (Appendix D) that provided an overview of substance misuse, produced by MU-ADEPT (the University of Missouri's Alcohol and Drug Education for Prevention and Treatment program) and funded by the Substance Abuse and Mental Health Services Administration (SAMHSA). Instructions for the preclass component were included in program-specific course syllabi and emailed to students prior to the in-person session (Appendix C).

In-person application session: The 2-hour live session consisted of three main components. The first component was viewing a brief video demonstration from SBIRT Colorado[9] (Appendix F) to review and consolidate knowledge gained from preclass preparation. Next was a peer role-play for students to practice SBIRT skills and share peer feedback (Appendices L-T). The third component was the SP interaction to apply skills and receive SP feedback (Appendices U-X). Faculty used the same logistics format (Appendices B and G), informational slides (Appendix H), and general script (Appendix I) at each in-person session.

Case Development

Case development followed three principles for success: connecting relevance to clinical practice,[10] faculty development for a shared IPE vision, and equality among all faculty participants.[11] All cases encompassed patients of varying ages,

Copyright © 2020 Clauser et al. This is an open-access publication distributed under the terms of the Creative Commons Attribution license.

medical settings, and reasons for seeking medical care. Alcohol use was the common theme for each scenario.

We adapted three cases for the peer role-play from existing SBIRT resources.[12] Each case contained a brief introduction, a summary of the setting and provider's role, background information for the patient's role including a completed screening questionnaire, and a feedback rubric for the observer role. The students worked in interprofessional triads; every student took a turn enacting each role (patient, provider, observer) over the course of three cases. Case roles were color coded by rounds so students could more easily follow the format (Appendices L-T). Students also received an agenda (Appendix K), copies of a screening tool (Appendix E), and an SBIRT pocket card (Appendix J).

For the SP interaction, each student remained in the same triad and rotated turns implementing the SBIRT process with the SP and providing peer-to-peer feedback. We adapted three cases for SPs based on SAMHSA training materials (Appendices U-X include two of these); each triad received one of the two patient backgrounds, which remained consistent. We created scripts of possible behavioral responses, which the SP changed for each of the three students in the triad. These responses included amicable to change, minimizing the problem, or resistant to change. All project faculty reviewed and edited each case for applicability across disciplines.

SP Training

For this project, an SP was defined as an individual trained to portray a real patient in order to simulate a set of symptoms or problems used for health care education.[13] All SPs were recruited and trained by the same experienced SP educator. As per the Association of Standardized Patient Educators' Standards of Best Practice recommendations, a psychologically and physically safe working environment was provided.[14] The SPs reviewed the scripts (Appendices U and V or W and X, depending on which case they were assigned) and attended an orientation and practice session at least 2 weeks prior to the activity. An additional training session occurred immediately prior to the activity and included final review of the script, as well as guidance on focused feedback methods. SPs were coached to start with an open-ended question to begin feedback (i.e., "How did you think it went?") followed by review of targeted feedback items from the student observer checklist (Appendix L) and ending with reflection on how they could collaborate with other professions with similar patients. SP training also included instructions about providing time-outs if students grew flustered or had an emotional reaction to the content. During the SP/student interactions, faculty rotated to each triad to observe interactions and troubleshoot if needed. Any suggestions for improving SP interactions with students were given to the SP educator, who communicated directly with the SPs. SPs were also provided an opportunity to debrief with the SP educator following each session. Suggestions for improvements were addressed by the faculty team at regularly scheduled planning and debriefing sessions.

Concluding the Session

After the SP interaction, students reconvened in the large group for debriefing. The debrief focused on the interprofessional collaborative experience (Appendix H, slide 7 notes). We asked students to complete an electronic survey at the end of the session.

Assessment of Student Performance and Perceptions

Program evaluation: All students were asked to complete a survey at the conclusion of the training (Appendix Y). The survey included 17 questions from the Center for Substance Abuse Training (as required by the SAMHSA grant), 10 questions from a modified Student Perceptions of Physician-Pharmacist Interprofessional Clinical Education (SPICE) tool,[3,15] and seven questions developed by our team. Two of the four Interprofessional Education Collaborative (IPEC) core competencies (Roles and Responsibilities, Teams and Teamwork) were addressed by the SPICE tool, so we added two questions regarding the remaining two IPEC core competencies (Communication and Collaboration, Values and Ethics).[16,17] One question asked students to rate SBIRT as an effective interprofessional learning activity, and one question addressed how useful the activity was to their interprofessional development.

Finally, students completed three open-ended qualitative questions:

1. Based on your experience, what do you believe are the benefits of an interprofessional team approach to caring for patients with substance abuse behaviors?
2. What about the training was most useful in supporting your work responsibilities?
3. How can Center for Substance Abuse Training improve its training?

We examined these responses qualitatively using principles of inductive reasoning—a common approach in qualitative analysis that allows researchers to gain insights beyond those possible through quantitative methods alone.[18] The lead qualitative

Copyright © 2020 Clauser et al. This is an open-access publication distributed under the terms of the Creative Commons Attribution license.

researcher monitored the process for quality and rigor by employing reflexivity and triangulation. Reflexivity acknowledges the presence of bias and seeks to mitigate its influence during the data-analysis process.[19,20] Triangulation is a methodological check on the validity of the data analysis and was accomplished by triangulating iterative coding phases among four qualitative researchers. Conceptual codes and subcodes[21] were developed as an outcome of this process.

Qualtrics Research Suite software was used to deploy the anonymous, web-based electronic survey. Quantitative data were summarized as frequencies (percentages) for each item.

Formative feedback: In addition to the feedback from SPs (described in SP Training, above), students provided peer-to-peer verbal feedback immediately following each role-play and SP interaction using the observational rubric (Appendix L). Observer forms were not collected or analyzed. The importance of giving specific constructive suggestions for improvement was reviewed with participants prior to the role-play activity. Faculty circulated and provided additional guidance on feedback where needed.

Results

Over 2 academic years from 2016 to 2018, 1,255 students participated in the interprofessional activity and completed any survey questions. Of these, 985 (79%) found the activity very useful/useful to their interprofessional development (learning objectives 1 and 4). Students were very satisfied/satisfied with the training regarding the following:

- The training enhanced my skills in this topic area (91%, learning objective 1).
- I expect to use the information gained from this training (91%, learning objectives 2 and 3).
- I expect this training to benefit my clients (93%, learning objective 3).

Students' perceived value of working with interprofessional teams was high following the training activity (Table 1).

Table 1. Students' Perceptions of the Value/Importance of Working With an Interprofessional Team Following an Interprofessional Training Activity Using the SBIRT Technique (*N* = 1,258)[a]

		Agree/Strongly Agree[c]		Neutral[c]		Disagree/Strongly Disagree[c]	
**Item[b]**	***N***	**No.**	**%**	**No.**	**%**	**No.**	**%**
Teams and teamwork							
Working with another discipline of students enhances my education.	1,253	1,177	94	67	5	9	1
Participating in educational experiences with another discipline of students enhances my future ability to work on an interdisciplinary team.	1,253	1,186	95	58	5	9	1
All health professions students should be educated to establish collaborative relationships with members from other disciplines.	1,252	1,210	97	41	3	1	0
Clinical simulations are the ideal place within their respective curricula for health professional students to interact.	1,252	1,144	91	96	8	12	1
Health professionals should collaborate in teams.	1,252	1,220	97	29	2	3	0
During their education, students from different health care disciplines should be involved in teamwork in order to understand their respective roles.	1,252	1,204	96	43	3	5	0
Roles and responsibilities							
My role within the interdisciplinary team is clearly defined.	1,254	1,022	81	178	14	54	4
I understand the roles of other professionals within the interdisciplinary team.	1,252	1,110	89	120	10	22	2
Patient and health outcomes							
Health outcomes are improved when patients are treated by a team of professionals from different disciplines.	1,255	1,217	97	36	3	2	0
Patient satisfaction is improved when patients are treated by a team of professionals from different disciplines.	1,254	1,190	95	61	5	3	0
IPEC core competencies[d]							
SBIRT is an effective activity for interprofessional learning.	1,253	1,141	91	79	6	33	3
SBIRT is an effective activity for developing mutual respect and shared value amongst a team of professionals from different disciplines. (VE)	1,253	1,150	92	86	7	17	1
SBIRT is an effective activity for developing skills to communicate with other health professionals, patients, families, or communities in a responsive and responsible manner. (CC)	1,254	1,148	92	87	7	19	2

Abbreviations: CC, Interprofessional Communication subcompetency; IPEC, Interprofessional Education Collaborative; SBIRT, screening, brief intervention, and referral to treatment; SPICE, Student Perceptions of Physician-Pharmacist Interprofessional Clinical Education tool; VE, Values/Ethics subcompetency.
[a]Students from doctor of nursing practice, medicine, pharmacy, social work, nutrition and exercise physiology, occupational therapy, and physician assistant programs of study were involved in training activities.
[b]A modified version of the SPICE tool was used to measure students' perceptions of the value and importance of working with an interprofessional team.
[c]Rated on a 5-point Likert scale (1 = *strongly agree,* 2 = *agree,* 3 = *neutral,* 4 = *disagree,* 5 = *strongly disagree*).
[d]Three questions evaluated students' perceptions about SBIRT as an effective activity for developing two of the four IPEC core competencies not addressed in the SPICE tool (CC, VE).

Copyright © 2020 Clauser et al. This is an open-access publication distributed under the terms of the Creative Commons Attribution license.

Qualitative data analysis resulted in seven themes across the three categories of usefulness (learning objectives 1 and 3), evaluation (learning objective 1), and benefit (learning objectives 2 and 4). Table 2 sets out the categories and their associated themes. Overall, students were highly satisfied with the interprofessional learning activity and interactions with an SP. Students believed this type of training prepared them to work collaboratively in teams and supported their comfort level in approaching difficult conversations with patients. For representative student comments arranged by category and theme, see Table 3.

A discipline-specific evaluation of student performance and perceptions of the potential impact of the training using a smaller cohort of students has been described elsewhere.[22]

Discussion

Growing interest in IPE activities stems from demonstrated improvements in patient safety[23] as well as accreditation requirements and the increasing recognition that health professional students benefit from engaging in cooperative work early in their training. We successfully used SBIRT as a model for SP simulation and realistic interprofessional collaboration.

We developed our cases with input and review from professionals in all seven participating disciplines; thus, all student participants were able to fully engage as equal members of the team in the SP interaction. The students learned about substance use and SBIRT from preclass preparation materials (Appendix D and SBIRT Oregon[7] materials) and had varying levels of prior experience with SPs. Students highly valued this SBIRT IPE learning experience as indicated by postexperience results.

Table 2. Students' Perceptions of the Value/Importance of an Interprofessional Team Learning Experience Using the SBIRT Technique

Category	Themes[a]
Training and usefulness to career-specific responsibilities	1. Value of the standardized patient in developing patient interviewing skills<br>2. Role-playing opportunities<br>3. Value of the tools provided by the faculty trainers to support the SBIRT learning activities
SBIRT program evaluation	1. A desire for more preexperience preparation specifically for the interactive aspect of the training session<br>2. An interest in expanding the variety of learning experiences included in the experience
Benefit of interprofessional learning related to substance use	1. Results in enhanced patient care<br>2. Improved understanding of the perspectives of others resulting in improved understanding of team member roles, thus supporting future collaborative interests

Abbreviation: SBIRT, screening, brief intervention, and referral to treatment.
[a]Theme perceptions were not stratified by profession in order to maintain anonymity of student responses.

The activity was very successful in students practicing interprofessional collaboration as well as the value of using a standardized process such as SBIRT. The vast majority of participants said that the activity enhanced their skills (91%), that they expected to use the information (93%), and that it would benefit their clients (93%). This activity gave students an opportunity to gain skills they found useful to their practice with current and future patients.

With three universities and seven disciplines involved, scheduling the interactive sessions was complex, but all disciplines successfully embedded the activity into an existing course. Interprofessional faculty collaboration was critical; this included early communication regarding logistics so each faculty could plan their courses accordingly, as well as regular meetings to coordinate the experience and then engage in ongoing assessment for program improvements. Numerous small-group sessions at varying times of day were needed to accommodate all disciplines because of highly diverse student schedules.

Strengths of this module include the focus on previsit interprofessional collaborative dialogue and planning, which allowed students to discuss and decide what each member's roles and responsibilities would be. Taking turns interviewing and counseling the SP helped develop each student's communication skills as well as the ability to observe other team members and provide real-time feedback using a rubric.

Potential limitations to this activity include the need for multiple health professions to be present, which may be a challenge, especially in more rural educational settings. SP simulations were done in triads, so this activity could be done with as few as two to three different health professions. In low-resource areas where programs may not have availability or funding for SP actors, faculty or students can use the scripts to play these roles. We included a videoconferencing option for students in rural educational sites; however, both students and faculty agreed that the technological frustrations outweighed the benefits of this component. A further limitation was that due to the small number of students in some professions, we did not identify themed perceptions by profession in order to protect participants' anonymity.

In future iterations of this activity, we would incorporate students describing their roles and those of other professions in screening

Copyright © 2020 Clauser et al. This is an open-access publication distributed under the terms of the Creative Commons Attribution license.

MedEdPORTAL® The AAMC Journal of Teaching and Learning Resources

Table 3. Students' Comments Regarding of the Value/Importance/Effectiveness of an Interprofessional Team Learning Experience Using the SBIRT Technique

Category and Themes	Student Comments
Usefulness to career-specific responsibilities	
Value of standardized patient	• "Learning about what to do when you are put into certain situations dealing with patients who may be displaying certain behaviors of alcohol use and possible abuse. Was good learning experience [because it] was very hands on and helps give you multiple perspectives."<br>• "I thought the training provided a valuable learning on how to communicate effectively with patients through different scenarios. Getting constructive feedback on how I counseled patients will help me polish up on my weaknesses in counseling. I also thought the impromptu scenario really helped me get a feel for the kind of interactions I will get in a real-life setting."<br>• "Before this training, I would not know where to start with this kind of conversation. I would feel too nervous to do it and would fear losing my patient's trust if I confronted them about drinking. This training reduce[d] my fear and discomfort. It helped give me a better idea of how patients' m[a]y feel about me talking to them about their alcohol use. I am really grateful to have this training."<br>• "The actor in the simulation was very realistic. She played the role of someone with a lifelong drinking problem and was very defiant and did not want help. This is something that, as a healthcare provider, I may come across and experience with my patients. Learning the right tools to communicate with her was key."<br>• "Learning how to effectively utilize motivational interviewing skills was most useful in supporting my work responsibilities. Specifically, I have always been turned off from being involved in patient care because I have not been good at helping patients who do not want my help. However, today I witnessed one of my teammates do an EXCELLENT job at communicating empathetically and compassionately with the standardized patient. This was very inspiring to me and I now have a goal to become a better motivational interviewer."
Value of peer role-plays	• "The feedback from peers [from] different programs [was valuable]."<br>• "Observing my peers was helpful because I was able to observe what was helpful and what I can do to improve my SBIRT [skills]."<br>• "Working in groups and having different views and opinions from other students in different fields helps you see your options in a certain circumstance."
Value of tools	• "As a future physician, I am sure that I will be dealing with patients regularly who are having trouble with substance abuse. Learning more about motivational interviewing and talking to patients about these issues, especially just bringing them up in the first place, will be extremely valuable in my career."<br>• "The videos [were useful] because they both provided new information that I was unaware of."<br>• "Giving us the [SBIRT] tool to evaluate and deliver meaningful feedback to our patients [with substance use] issues [was valuable]."<br>• "I enjoyed the online training and learned a lot from it. I would have enjoyed more live or video examples."<br>• "The [SBIRT] reference card was helpful."
SBIRT program evaluation	
Preparation	• "I think that it would have been helpful to have more of a review of the SBIRT concepts before we jumped right into practice. I felt unprepared at first, but after practicing I definitely feel more prepared to speak with an actual patient."<br>• "Technological difficulties took time away from practicing role plays and hindered the overall learning experience."<br>• "The training was not well organized for the distant site. We were not able to use the time as effectively because of that."<br>• "Make these much more common! If we could all get training like this more often we would be SO much better set for real life and actually improving the lives of our patients that we see every day."
Variety of learning experiences	• "This training could be improved by emphasizing why this training is important to our related field."<br>• "Tailor more towards each individual profession."<br>• "It's hard to envision a time when this would be used in a retail pharmacy setting, so offering examples of that use would be beneficial."<br>• "Providing resources [locally] that we could give to our clients—that will increase our knowledge of resources as well as help us practice."<br>• "More training on how to refer a patient to treatment or offer other services."<br>• "How to offer more to the patient after doing the SBIRT part."<br>• "It would be great to get copies of the guidelines and materials for future reference."<br>• "I'd be interested in a little information on how this technique and training creates change—numbers or stories of its effectiveness."<br>• "Otherwise, it was a great introduction to the topics and skills."
Benefit of interprofessional approach to learning about SBIRT	
Enhanced patient care	• "I believe working together will give better patient outcomes every time. The patient will know that multiple people care about their health, which will motivate them to change."<br>• "[SBIRT helps ensure] the patient is not lost within the healthcare system. It allows and ensures that follow up is taking place to help support the 'whole' person… physical, mental, emotional & spiritual."
Value of perspectives	• "[It was valuable] facing challenges as a group, and listening to different viewpoints."<br>• "Each team member sees only one snippet of an individual's life. Working collaboratively would be effective because you would understand the client and their needs."<br>• "Everyone has their strengths, [which may be] why they are in different programs. Being with a student [from another discipline] helped me learn how to be an effective motivational interviewer because of how GREAT she was at effectively counseling the patient."<br>• "Every person comes from a different background and brings a whole new perspective and wealth of knowledge to these situations. It's great getting to interact with other students who have a depth of knowledge in something I know nothing about. It allows us to teach each other and grow our interprofessional relationship organically."

Abbreviation: SBIRT, screening, brief intervention, and referral to treatment.

Copyright © 2020 Clauser et al. This is an open-access publication distributed under the terms of the Creative Commons Attribution license.

for substance misuse during the icebreaker activity (Appendix H, slide 2). This activity represents an SP simulation that can be used by any program that desires to have a high-quality, single-session, collaborative interprofessional experience, with special focus on substance use screening and motivational interviewing.

Appendices

A. Educational Objectives.docx
B. Administrative Instructions Prior to Session.docx
C. Student Overview of SBIRT Components - Email Prior.docx
D. Student Prep - ADEPT Video.mp4
E. AUDIT Screening Tool - Email and Print.docx
F. Demonstration - SBIRT Colorado.mp4
G. Faculty Overview and Agenda.docx
H. SBIRT Slides for Live Session.pptx
I. Faculty Script for Slide Presentation.docx
J. SBIRT Pocket Card - Print.pdf
K. Student Agenda - Print.docx
L. Peer Role-Play Case 1-Print ORANGE-Observer.docx
M. Peer Role-Play Case 1-Print ORANGE-Patient.docx
N. Peer Role-Play Case 1-Print ORANGE-Provider.docx
O. Peer Role-Play Case 2-Print BLUE-Observer.docx
P. Peer Role-Play Case 2-Print BLUE-Patient.docx
Q. Peer Role-Play Case 2-Print BLUE-Provider.docx
R. Peer Role-Play Case 3-Print GREEN-Observer.docx
S. Peer Role-Play Case 3-Print GREEN-Patient.docx
T. Peer Role-Play Case 3-Print GREEN-Provider.docx
U. SP Case Jamie Quimby.docx
V. SP AUDIT Screen Jamie Quimby.pdf
W. SP Case Pat Stewart.docx
X. SP AUDIT Screen Pat Stewart.pdf
Y. Evaluation Tool.docx

All appendices are peer reviewed as integral parts of the Original Publication.

Janelle Clauser, MD: Clinical Associate Professor, Department of Internal Medicine, University of Washington School of Medicine; ORCID: https://orcid.org/0000-0002-5614-8245

Barbara B. Richardson, PhD, RN: Clinical Associate Professor, Department of Medical Education and Clinical Sciences, Elson S. Floyd College of Medicine, Washington State University

Tamara Odom-Maryon, PhD: Research Professor, College of Nursing, Washington State University

Donna Mann, OTD, MEd: Associate Dean, College of Health Science and Public Health, and Associate Professor, Department of Occupational Therapy, Eastern Washington University

Megan N. Willson, PharmD: Clinical Associate Professor, Department of Pharmacotherapy, College of Pharmacy and Pharmaceutical Sciences, Washington State University

Patricia L. Hahn, MN, ARNP: Senior Lecturer, Department of Family Medicine, University of Washington School of Medicine

Janet Purath, PhD, ARNP: Associate Professor, College of Nursing, Washington State University

Erica Tuell: Research Study Coordinator, College of Nursing, Washington State University

Catrina R. Schwartz, PharmD: Pharmacist, Pharmacy Management, Medication Review, Inc.

Dawn DePriest, DNP, ARNP: Clinical Assistant Professor, College of Nursing, Washington State University

Disclosures
None to report.

Funding/Support
This project was funded by the Substance Abuse and Mental Health Services Administration, grant number 5H79T1026015, FAIN: T1026015.

Prior Presentations
Purath J, Fincham S, Odom-Maryon T. Teaching SBIRT to interprofessional groups of students. Presented at: American Association of Nurse Practitioners Conference; March 2018; Denver, CO.

Clauser J, Richardson B, Mann D, Bray B. Multi-institution collaboration: creating and evaluating a standardized patient-based interprofessional learning session using an established substance use tool (SBIRT). Poster presented at: Group on Regional Medical Campuses Spring Meeting; April 15-17, 2019; Spokane, WA.

Kobayashi R, Schwartz C, Willson M, et al. Interprofessional student training: an evaluation of teaching screening, brief intervention and referral to treatment (SBIRT). *J Soc Work Pract Addict*. 2019;19(1-2):26-46. https://doi.org/10.1080/1533256X.2019.1589882

Ethical Approval
Reported as not applicable.

References

1. King S, Hall M, McFarlane L, et al. Launching first-year health sciences students into collaborative practice: highlighting institutional enablers and barriers to success. *J Interprof Care*. 2017;31(3):386-393. https://doi.org/10.1080/13561820.2016.1256870

Copyright © 2020 Clauser et al. This is an open-access publication distributed under the terms of the Creative Commons Attribution license.

2. *Framework for Action on Interprofessional Education & Collaborative Practice*. World Health Organization; 2010.
3. Ward LD, Bray BS, Odom-Maryon TL, et al. Development, implementation and evaluation of a longitudinal interprofessional education project. *J Interprof Educ Pract*. 2016;3:35-41. https://doi.org/10.1016/j.xjep.2016.04.003
4. Wamsley M, Treit K, Satterfield J, et al. Three standardized patient cases to measure screening, brief intervention and referral to treatment (SBIRT) skills in primary care residents. *MedEdPORTAL*. 2011;7:9005. https://doi.org/10.15766/mep_2374-8265.9005
5. Pitts S, Borus J, Harris S, et al. Benefitting Brad: an interprofessional case of adolescent substance use and ADHD. *MedEdPORTAL*. 2015;11:10224. https://doi.org/10.15766/mep_2374-8265.10224
6. Soskin P, Duong D. Social worker as SBIRT instructor to emergency medicine residents. *MedEdPORTAL*. 2014;10:9840. https://doi.org/10.15766/mep_2374-8265.9840
7. Winkle J. SBIRT in medical settings. SBIRT Oregon. Accessed June 2019. https://www.sbirtoregon.org/wp-content/uploads/SBIRT-in-medical-settings-pdf-1.pdf
8. Resources—ADEPT training materials—featured videos: Intro to ADEPT. MU ADEPT. Accessed June 2019. https://adept.missouri.edu/resources/
9. Adult SBIRT screening tools: Brief Intervention Demonstration Video. Peer Assistance Services. Accessed August 2019. www.sbirtcolorado.org/adult-sbirt-screening-tools
10. Hammick M, Freeth D, Koppel I, Reeves S, Barr H. A best evidence systematic review of interprofessional education: BEME Guide no. 9. *Med Teach*. 2007;29(8):735-751. https://doi.org/10.1080/01421590701682576
11. Lawlis TR, Anson J, Greenfield D. Barriers and enablers that influence sustainable interprofessional education: a literature review. *J Interprof Care*. 2014;28(4):305-310. https://doi.org/10.3109/13561820.2014.895977
12. Horowitz B. Learning to teach SBIRT, practicing what we preach. Presented at: SBIRT Grantee Conference; March 21-23, 2016; Washington, DC.
13. Lioce L, Lopreiato J, Downing D, et al.; Terminology and Concepts Working Group, eds. *Healthcare Simulation Dictionary*. 2nd ed. Agency for Healthcare Research and Quality; 2020. AHRQ publication no. 20-0019.
14. Lewis KL, Bohnert CA, Gammon WL, et al. The Association of Standardized Patient Educators (ASPE) Standards of Best Practice (SOBP). *Adv Simul (Lond)*. 2017;2:10. https://doi.org/10.1186/s41077-017-0043-4
15. Fike DS, Zorek JA, MacLaughlin AA, Samiuddin M, Young RB, MacLaughlin EJ. Development and validation of the Student Perceptions of Physician-Pharmacist Interprofessional Clinical Education (SPICE) instrument. *Am J Pharm Educ*. 2013;77(9):190. https://doi.org/10.5688/ajpe779190
16. Interprofessional Education Collaborative Expert Panel. *Core Competencies for Interprofessional Collaborative Practice: Report of an Expert Panel*. Interprofessional Education Collaborative; 2011.
17. Interprofessional Education Collaborative Expert Panel. *Core Competencies for Interprofessional Collaborative Practice: 2016 Update*. Interprofessional Education Collaborative; 2016.
18. Dillaway H, Lysack C, Luborsky MR. Qualitative approaches to interpreting and reporting data. In: Kielhofner G. *Research in Occupational Therapy: Methods of Inquiry for Enhancing Practice*. FA Davis; 2006:372-388.
19. Clancy M. Is reflexivity the key to minimising problems of interpretation in phenomenological research? *Nurse Res*. 2013;20(6):12-16. https://doi.org/10.7748/nr2013.07.20.6.12.e1209
20. Darawsheh W, Stanley M. Reflexivity in research: promoting rigour, reliability and validity in qualitative research. *Int J Ther Rehabil*. 2014;21(12):560-568. https://doi.org/10.12968/ijtr.2014.21.12.560
21. Bradley EH, Curry LA, Devers KJ. Qualitative data analysis for health services research: developing taxonomy, themes, and theory. *Health Serv Res*. 2007;42(4):1758-1772. https://doi.org/10.1111/j.1475-6773.2006.00684.x
22. Kobayashi RIE, Schwartz CR, Willson MN, et al. Interprofessional student training: an evaluation of teaching screening, brief intervention and referral to treatment (SBIRT). *J Soc Work Pract Addict*. 2019;19(1-2):26-46. https://doi.org/10.1080/1533256X.2019.1589882
23. Gupta B, Nanda A, Jain V, Verma M. Interprofessional education: a reform plan for collaborative. *Contemp Clin Dent*. 2017;8(1):3-6. https://doi.org/10.4103/0976-237X.205035

Received: October 29, 2019
Accepted: February 12, 2020
Published: September 11, 2020

Copyright © 2020 Clauser et al. This is an open-access publication distributed under the terms of the Creative Commons Attribution license.
